# Supplementary material for: Chondroitin sulfate proteoglycans prevent immune cell phenotypic conversion and inflammation resolution via TLR4 in rodent models of spinal cord injury
Source: Nat Commun. 2022 May 25;13:2933. doi: 10.1038/s41467-022-30467-5 (PMC9133109; doi:10.1038/s41467-022-30467-5)
Supplement: Supplementary file 1 — Supplementary Information [file 41467_2022_30467_MOESM1_ESM.pdf]

Chondroitin sulfate proteoglycans prevent immune cell phenotypic conversion and inflammation resolution via TLR4 in rodent models of spinal cord injury

Isaac Francos-Quijorna[1†], Marina Sánchez-Petidier[2*], Emily R. Burnside[1†*], Smaranda R. Badea[1*], Abel Torres-Espin[3], Lucy Marshall[1], Fred de Winter[4], Joost Verhaagen[4,5], Victoria Moreno-Manzano[2], Elizabeth J. Bradbury[1#]

SUPPLEMENTARY MATERIAL

Supplementary Figures

Legends for Supplementary Data

Supplementary Tables

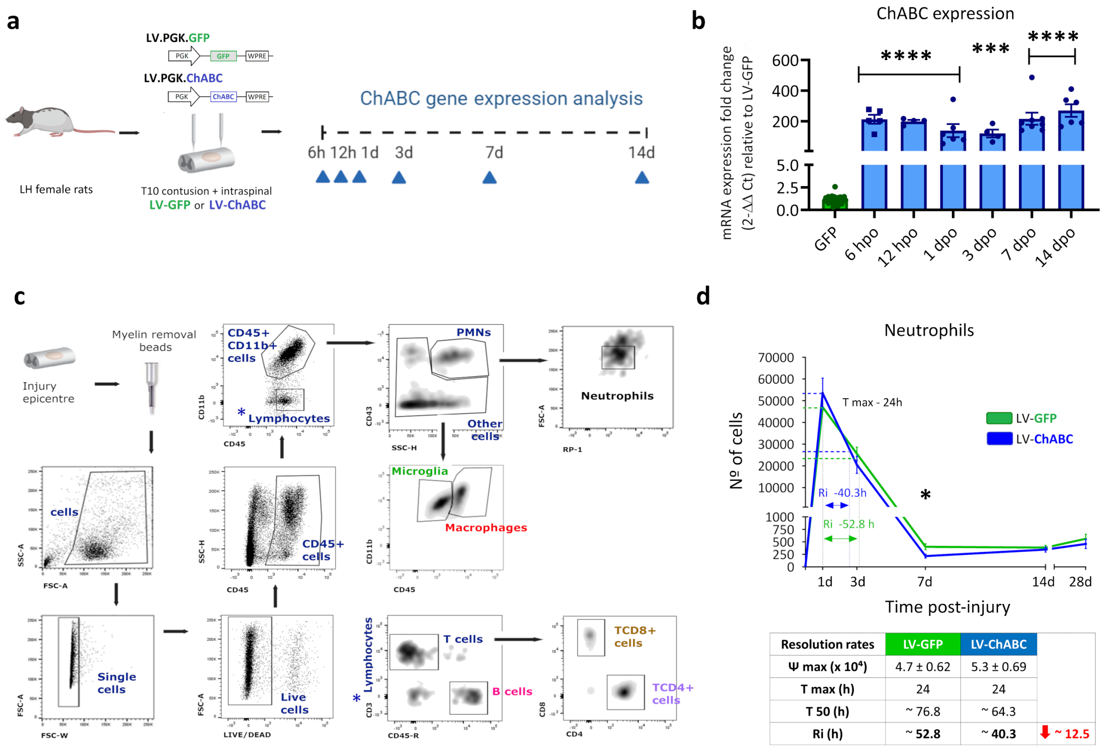

Supplementary Figure 1. Lentiviral ChABC treatment enables high ChABC transgene expression in the injured spinal cord and modulates immune cell responses after spinal cord injury. (a) Experimental design of ChABC expression analysis. (b) ChABC gene expression measured by qPCR on RNA extracted from the injury epicentre and injection site at different timepoints after SCI. **p < 0.01, ****p < 0.0001 versus control group (LV-GFP). Results were assessed for normality using the Shapiro-Wilk test and one-way ANOVA with Dunnett post hoc test was used to analyse group differences (n = 31 for LV-GFP treatment group, n = 5 for LV-ChABC treatment group at 6 hpi; n= 4 at 12 hpi; n= 6 at 1 dpi; n = 4 at 3 dpi; n = 8 at 7 dpi; n = 6 at 14 dpi). Data are shown as mean ± SEM. (c) Manual gating strategy used for immune cell recruitment assessment. (d) Graph showing the quantification of neutrophil recruitment and table with its resolution rates following spinal cord injury at 1, 3, 7, 14 and 28 dpi with (LV-ChABC) or without (LV-GFP) CSPG digestion. *p < 0.05 versus control (LV-GFP) group. Results were assessed for normality using the Shapiro-Wilk test and analysed using a two-way ANOVA with Bonferroni's post hoc test (n = 8 at 1 dpi; n = 11 at 3 dpi; n = 10 at 7 dpi; n = 6 at 14 dpi; n = 4 at 28 dpi per each treatment group). Data are pooled from at least two independent experiments. Data are shown as mean ± SEM.

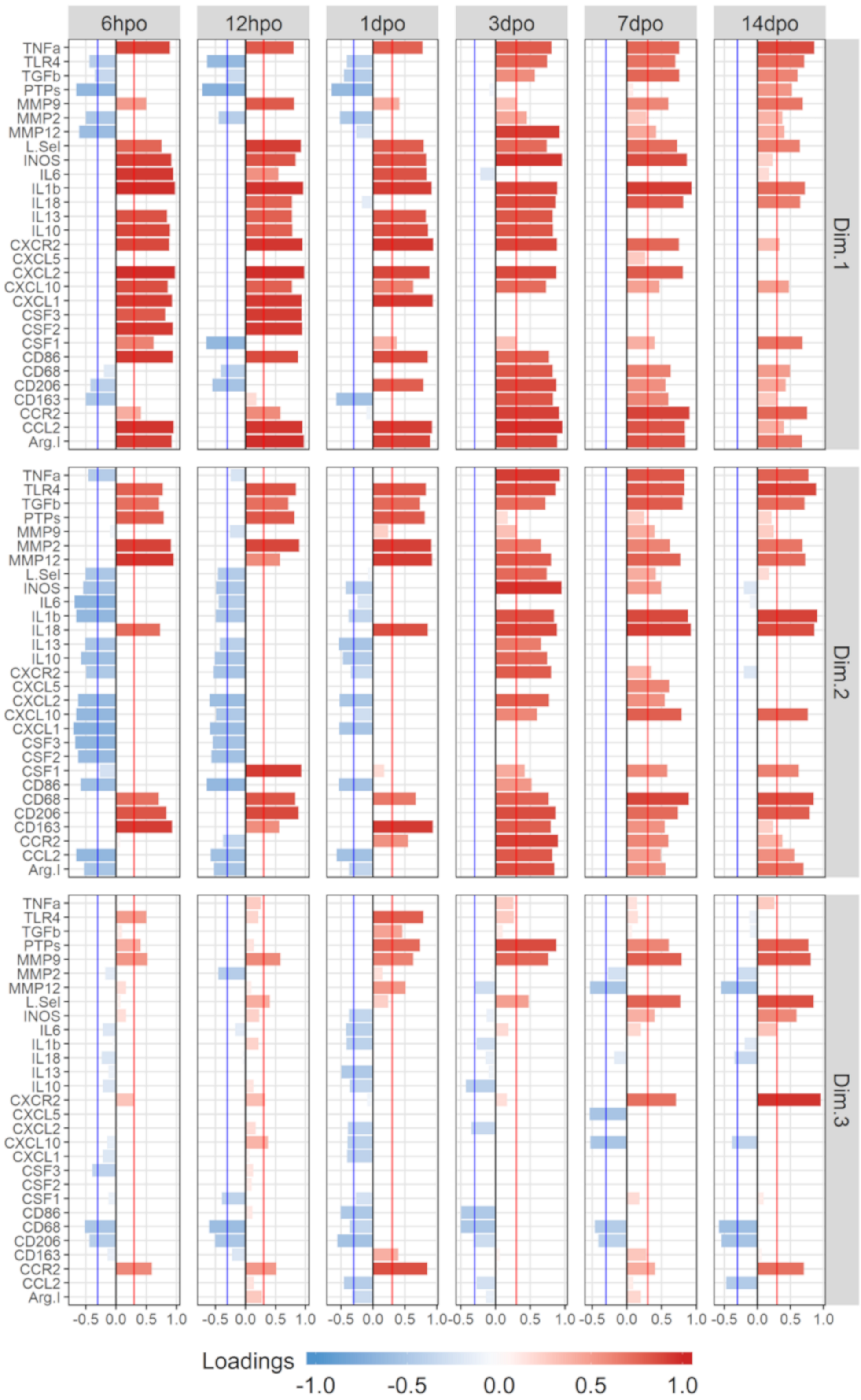

Supplementary Figure 2. Inflammatory gene co-expression pattern over time. Correlated loadings for each of the 3 dimensions of cytokine co-expression represented in Fig. 2. Changes over time showing the inflammatory gene co-expression that contributes to each pattern.

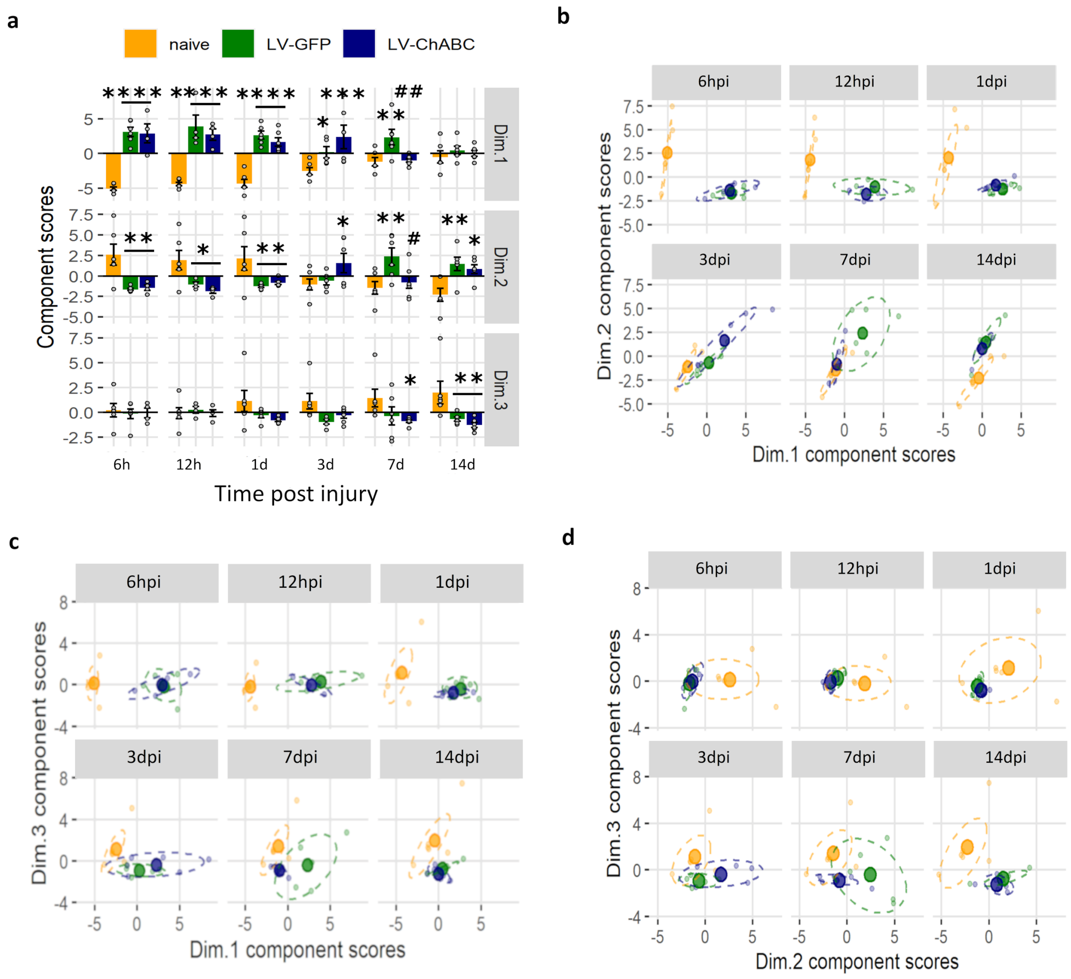

Supplementary Figure 3. Dynamic shift over 3 inflammatory co-expression patterns. (a-d) The multivariate co-expression patterns shown in Fig. 2 are expanded here. (a) Component scores bar graph representation for each group and dimension over time. A dynamic shift was revealed in dimension 1 and 2 between naïve and LV injected animals, and between LV-GFP and LV-ChABC treatment at 7 dpi. Dimension 3 captured a pattern of co-expression diverging between naïve and LV-injected animals at later time-points. Two-way ANOVA with time and group as factors using Tukey for multiple testing correction. *p < 0.05, **p < 0.01, ***p < 0.001 versus uninjured (naive) group; #p < 0.05, ###p < 0.001 versus LV-GFP group. Data are shown as mean ± SEM (n is number of animals/samples where n = 6 for each group-time combination except LV-GFP at 12h, LV-ChABC at 6h and 12h post injury (n = 4); LV-GFP at 3 dpi (n = 5) and LV-ChABC at 7 dpi (n = 7). Data are shown as mean ± SEM. (b-d) Bidimensional representations of component scores dim. 1 vs. dim. 2 (b), dim. 1 vs. dim. 3 (c) and dim. 2 vs. dim. 3 (d). Ellipsoids represent the bivariate standard deviation and the coloured circles the centroid.

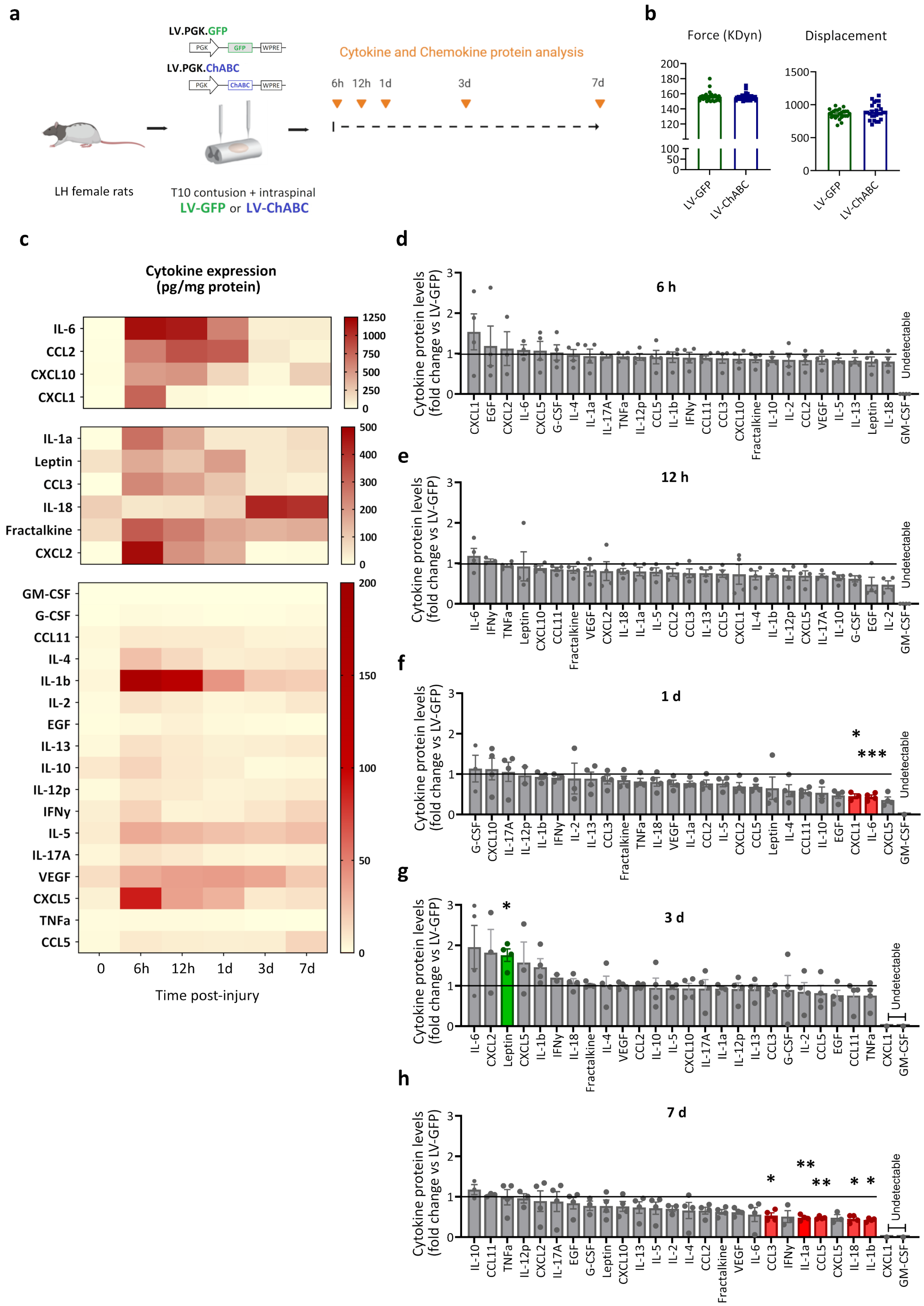

a
LV.PGK.GFP
LV.PGK.ChABC
Cytokine and Chemokine protein analysis
6h 12h 1d 3d 7d
LH female rats
T10 contusion + intraspinal
LV-GFP or LV-ChABC
b
Force (KDyn)
Displacement
LV-GFP
LV-ChABC
c
Cytokine expression
(pg/mg protein)
IL-6
CCL2
CXCL10
CXCL1
IL-1a
Leptin
CCL3
IL-18
Fractalkine
CXCL2
GM-CSF
G-CSF
CCL11
IL-4
IL-1b
IL-2
EGF
IL-13
IL-10
IL-12p
IFNγ
IL-5
IL-17A
VEGF
CXCL5
TNFa
CCL5
0 6h 12h 1d 3d 7d
Time post-injury
d
6 h
e
12 h
f
1 d
g
3 d
h
7 d
Cytokine protein levels
(fold change vs LV-GFP)
Undetectable

Supplementary Figure 4. CSPG digestion reduces inflammatory cytokine expression during the resolution phase of inflammation. (a) Experimental design of cytokine and chemokine expression analysis. (b) Contusion device impact force and displacement measurements confirm reproducible and consistent injuries between treatment groups. Data were determined as normally distributed by the Shapiro-Wilk test and subsequently analysed using a two-tailed unpaired t test. n = 26 animals per treatment group. Data are shown as mean ± SEM. (c) Cytokine protein level profile in control group (LV-GFP) at different time points after SCI. (d-h) Cytokine protein level comparison between LV-GFP and LV-ChABC treatment groups assessed by Luminex analysis at different time-points post injury. (h) CSPG digestion significantly reduced the expression of CCL3, CCL5, IL-18, IL1a and IL1b (red bars) compared with control treatment (LV-GFP) at 7 dpi. *$p < 0.05$, **$p < 0.01$, ***$p < 0.001$, ****$p < 0.0001$ versus control (LV-GFP) group. (d-h) Results were assessed for normality using the Shapiro-Wilk test and analysed using a two-tailed unpaired t test. Data are shown as mean ± SEM (n = 3 in naïve animals, n = 4 per treatment group and time-point except 3dpi where n = 3 per treatment).

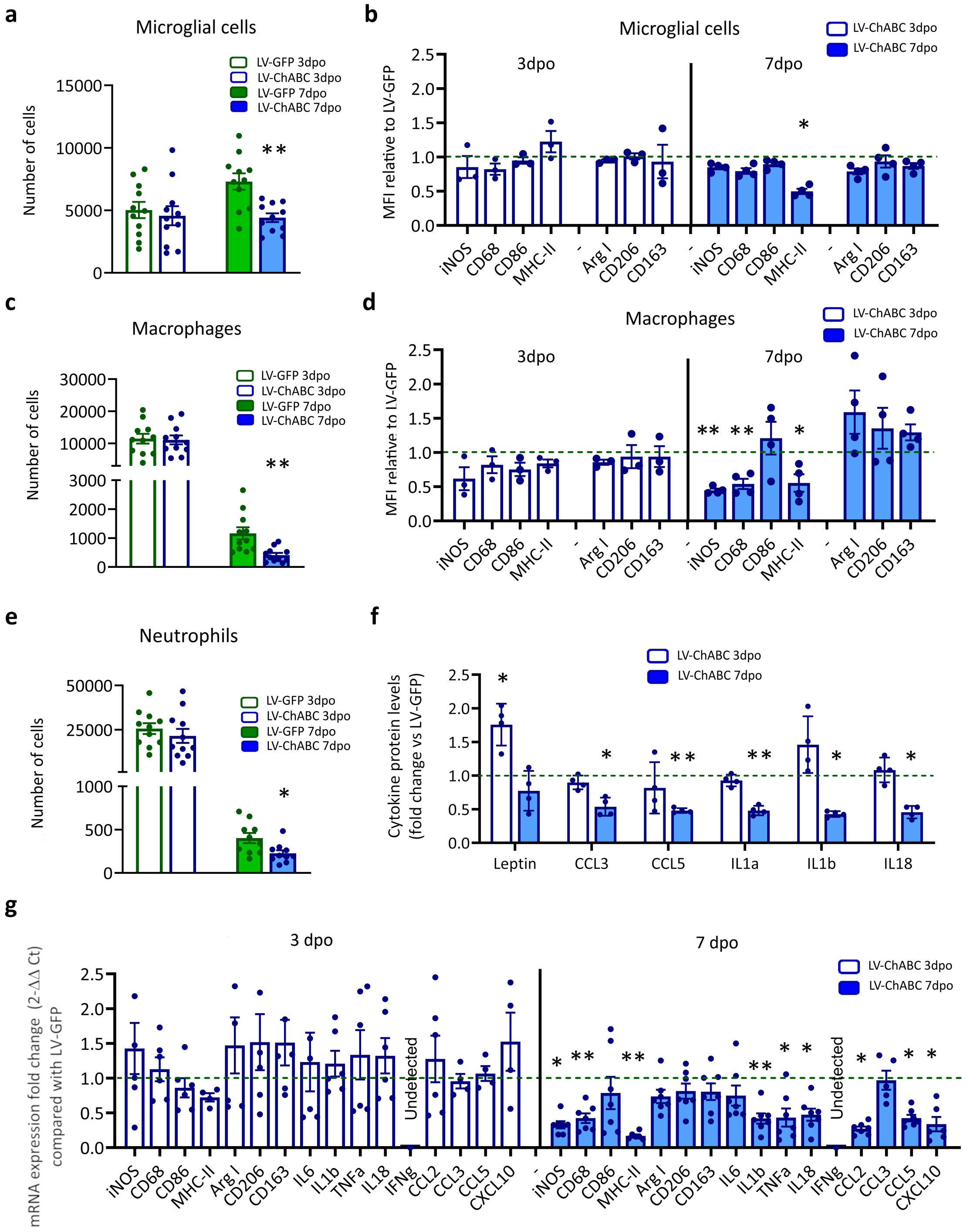
a
Microglial cells
LV-GFP 3dpo
LV-ChABC 3dpo
LV-GFP 7dpo
LV-ChABC 7dpo
Number of cells
b
Microglial cells
3dpo
7dpo
MFI relative to LV-GFP
iNOS
CD68
CD86
MHC-II
Arg I
CD206
CD163
c
Macrophages
d
Macrophages
e
Neutrophils
f
Cytokine protein levels (fold change vs LV-GFP)
Leptin
CCL3
CCL5
IL1a
IL1b
IL18
g
3 dpo
7 dpo
mRNA expression fold change (2-ΔΔ Ct) compared with LV-GFP
IL6
TNFa
IFNg
CCL2
CXCL10
Undetected

Supplementary Figure 5. 7 dpi is the earliest time-point when CSPG digestion significantly modulates multiple aspects of the inflammatory response after SCI. Bar graphs showing cell number within the injured spinal cord at 3 dpi and 7 dpi in (a) microglial cells, (c) monocyte/macrophages and (e) neutrophils. (b) Bar graph comparing relative changes in the expression of classic M1-like and M2-like markers in microglial cells. (d) Bar graph comparing relative changes in the expression of classic M1-like and M2-like markers in monocyte/macrophages. Microglial cell, monocyte/macrophage and neutrophil cell numbers are decreased at 7 dpi only and microglial cells and monocytes have significantly reduced expression of M1-like markers in the LV-ChABC treated group compared to LV-GFP at 7dpi. (a, c, e) Data extracted from samples in Fig 1 a-e. *$p < 0.05$, **$p < 0.01$ versus control (LV-GFP) group. Results were assessed for normality using the Shapiro-Wilk test and analysed using a two-tailed unpaired t test. Data are shown as mean ± SEM ($n = 11$ per treatment and time-point). Data are pooled from at least two independent experiments. (b, d) Data extracted from samples in Fig 3 a-h. *$p < 0.05$ **$p < 0.01$ versus control (LV-GFP) group. Results were assessed for normality using the Shapiro-Wilk test and analysed using a two-tailed unpaired t test. Data are shown as mean ± SEM ($n = 4$ per treatment at 3dpi and $n = 4$ per treatment at 7dpi). MFI, mean fluorescence intensity. (f) Cytokine protein level comparison between LV-GFP and LV-ChABC treatment groups assessed by Luminex analysis at 3- and 7-days post injury. Data extracted from samples in Extended Fig 4. Results were assessed for normality using the Shapiro-Wilk test and analysed using a two-tailed unpaired t test. *$p<0.05$, **$p < 0.01$ versus control (LV-GFP) group. Data are shown as mean ± SEM ($n = 2$ in naïve animals, $n = 4$ per treatment group and time-point. (g) Bar graph showing inflammatory related gene expression differences at the injured epicentre between LV-GFP and LV-ChABC treatments at 3 and 7 dpi. *$p < 0.05$, **$p < 0.01$ versus control (LV-GFP) group. Results were assessed for normality using the Shapiro-Wilk test and analysed using a two-tailed unpaired t test. Data are shown as mean ± SEM ($n = 6$ per treatment at 3dpi and $n = 7$ per treatment at 7dpi).

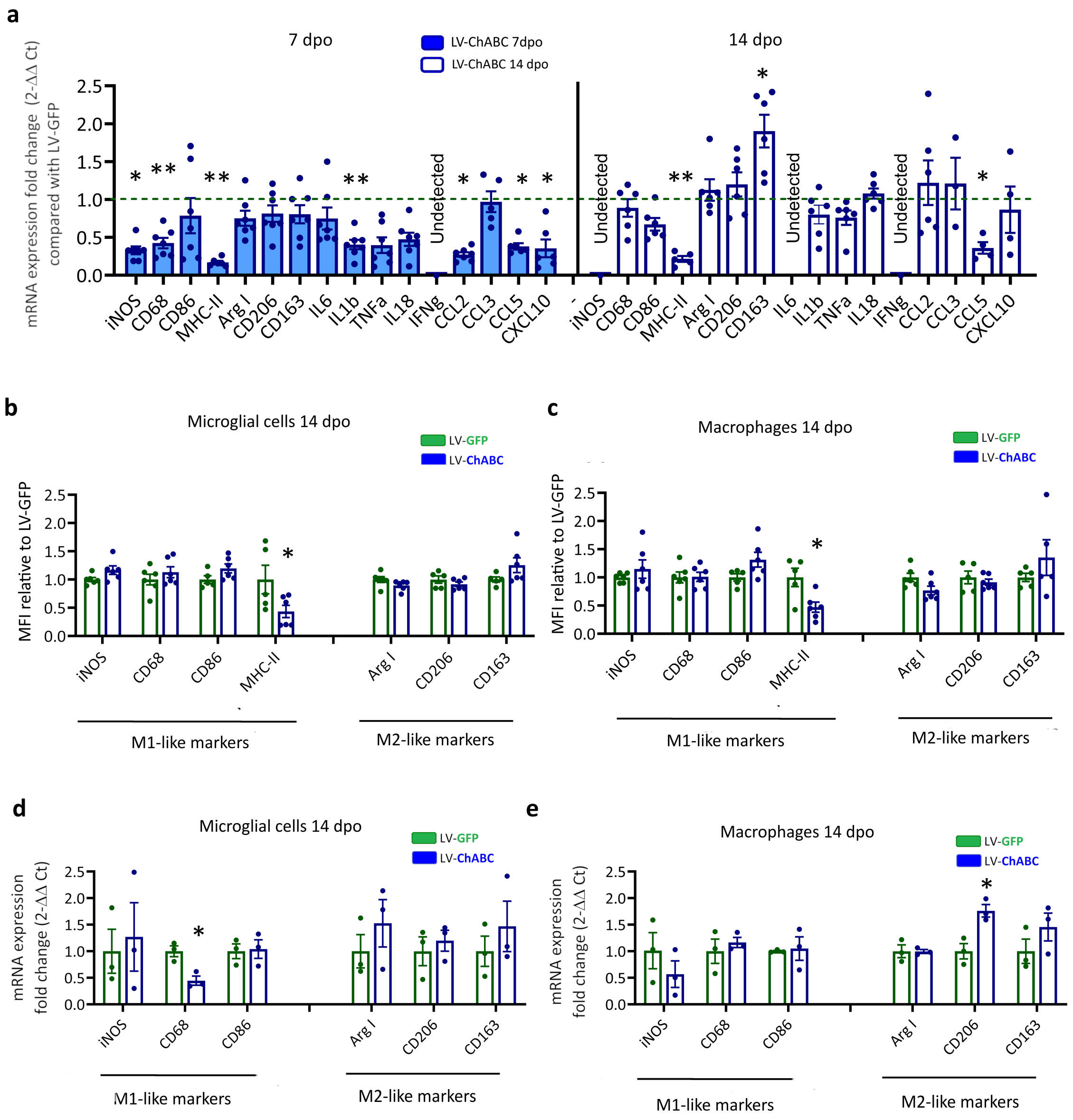
a
7 dpo
14 dpo
LV-ChABC 7dpo
LV-ChABC 14 dpo
mRNA expression fold change (2-ΔΔ Ct) compared with LV-GFP
Undetected
iNOS
CD68
CD86
MHC-II
Arg I
CD206
CD163
IL6
IL1b
TNFa
IL18
IFNg
CCL2
CCL3
CCL5
CXCL10
b
Microglial cells 14 dpo
c
Macrophages 14 dpo
LV-GFP
LV-ChABC
MFI relative to LV-GFP
M1-like markers
M2-like markers
d
Microglial cells 14 dpo
e
Macrophages 14 dpo
mRNA expression fold change (2-ΔΔ Ct)

Supplementary Figure 6. Inflammatory resolution effect of CSPG digestion is maintained in microglia and infiltrating monocytes/macrophages at 14 dpi. (a) Bar graph showing significant pro-inflammatory gene expression differences at the injured epicentre between LV-GFP and LV-ChABC treatments at 7 and 14 dpi. mRNA levels assessed by qPCR. Results were assessed for normality using the Shapiro-Wilk test and analysed using a two-tailed unpaired t test. Data are shown as mean ± SEM. (n = 7 per treatment at 7dpi and n = 6 per treatment at 14dpi). *p < 0.05, **p < 0.01 versus control (LV-GFP) group. Bar graphs showing changes in the expression of classic M1-like and M2-like signature markers assessed by MFI (mean fluorescence intensity) in (b) microglial cells and (c) monocytes/macrophages at 2 weeks after SCI. The reduction of MHC-II exhibited at 7dpi by both cell populations following CSPG digestion is maintained. (b-c) Results were assessed for normality using the Shapiro-Wilk test and analysed using a two-tailed unpaired t test. Data are shown as mean ± SEM. (n = 6 per treatment and cell type). *p < 0.05 versus control (LV-GFP) group. Gene expression of classic M1-like and M2-like phenotype markers measured by qPCR in sorted microglial cells (d) and monocytes/macrophages (e) LV-ChABC treatment reduced M1-like CD68 expression in microglial cells 14 dpi after SCI. (d-e) Results were assessed for normality using the Shapiro-Wilk test and analysed using a two-tailed unpaired t test. Data are shown as mean ± SEM. (n = 3 per treatment and cell type). *p < 0.05 versus control (LV-GFP) group.

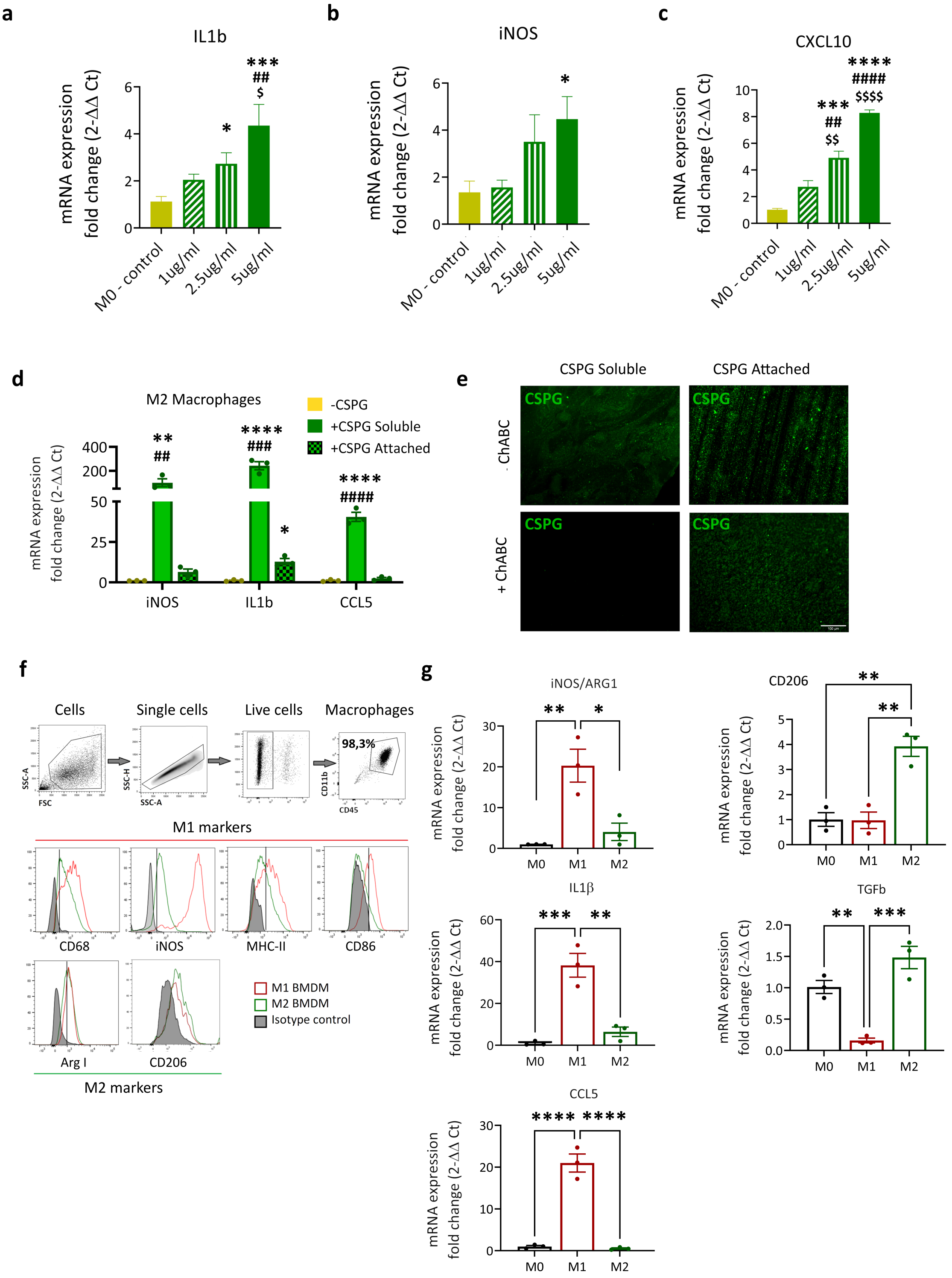
a
IL1b
mRNA expression
fold change (2-ΔΔ Ct)
***
##
$
*
M0 - control
1ug/ml
2.5ug/ml
5ug/ml
b
iNOS
mRNA expression
fold change (2-ΔΔ Ct)
*
M0 - control
1ug/ml
2.5ug/ml
5ug/ml
c
CXCL10
mRNA expression
fold change (2-ΔΔ Ct)
****
####
$$$$
***
##
$$
M0 - control
1ug/ml
2.5ug/ml
5ug/ml
d
M2 Macrophages
-CSPG
+CSPG Soluble
+CSPG Attached
mRNA expression
fold change (2-ΔΔ Ct)
**
##
****
###
****
####
*
iNOS
IL1b
CCL5
e
CSPG Soluble
CSPG Attached
- ChABC
+ ChABC
CSPG
f
Cells
Single cells
Live cells
Macrophages
98,3%
SSC-A
FSC
SSC-H
CD11b
CD45
M1 markers
CD68
iNOS
MHC-II
CD86
Arg I
CD206
M2 markers
M1 BMDM
M2 BMDM
Isotype control
g
iNOS/ARG1
mRNA expression
fold change (2-ΔΔ Ct)
**
*
M0
M1
M2
CD206
**
**
IL1β
***
**
TGFb
**
***
CCL5
****
****

Supplementary Figure 7. Optimization of bone marrow derived macrophage (BMDM) CSPG treatment in vitro. (a-c) Effect of CSPG treatment on key pro-inflammatory gene expression in non-polarized (M0) BMDMs: dose response. Bar graphs show (a) Il1b, (b) iNOS and (c) CXCL10 mRNA expression, determined by qPCR. Results were assessed for normality using the Shapiro-Wilk test and one-way ANOVA with Tukey(-Kramer) post hoc test was used to analyse group differences. *$p < 0.05$, **$p < 0.01$, ***$p < 0.001$, ****$p < 0.0001$ versus control (M0), or # versus 1 ug/ml group, $ versus 2.5 ug/ml group. Relative fold changes were compared to the control group and are presented as mean ± SEM (n = 5 control group, n = 3 for each CSPG concentration treatment). (d) Bar graphs showing expression of inflammatory genes, comparing soluble CSPG treatment with CSPGs attached on coated PDL at the same concentration (5 ug/ml). mRNA levels of inflammatory response genes were determined by qPCR. *$p < 0.05$, **$p < 0.01$, ***$p < 0.001$ soluble CSPGs vs control group (without CSPGs); #$p < 0.05$, ##$p < 0.01$, ###$p < 0.001$ attached CSPGs vs control group (without CSPGs). Results were assessed for normality using the Shapiro-Wilk test and one-way ANOVA with Tukey post hoc test used to analyse group differences. Data are shown as mean ± SEM (n = 3 per group). (e) ICC of anti-CSPG (green) on PDL coating before (-CSPG) and after (+CSPG) CSPG digestion, demonstrating that only CSPGs in the soluble condition are accessible to digestion by ChABC. Experiment was repeated twice independently with similar results. (f) Gating strategy used to evaluate survival, purity and phenotype marker expression of BMDMs 24 h after polarization (top). FACS plot histogram of pro-inflammatory (middle) and anti-inflammatory (bottom) markers on cultured BMDM. Red and green lines represent M1 and M2 polarized BMDMs, respectively. Grey colour represents the isotype controls. (g) Gene expression of pro-inflammatory and anti-inflammatory markers in polarized and non-polarized BMDMs assessed by qPCR. Compared to the control group (non-polarized or M0), relative fold changes are presented as mean ± SEM. Results were assessed for normality using the Shapiro-Wilk test and one-way ANOVA with Tukey post hoc test was used to analyse significant differences. (n = 3 per group).

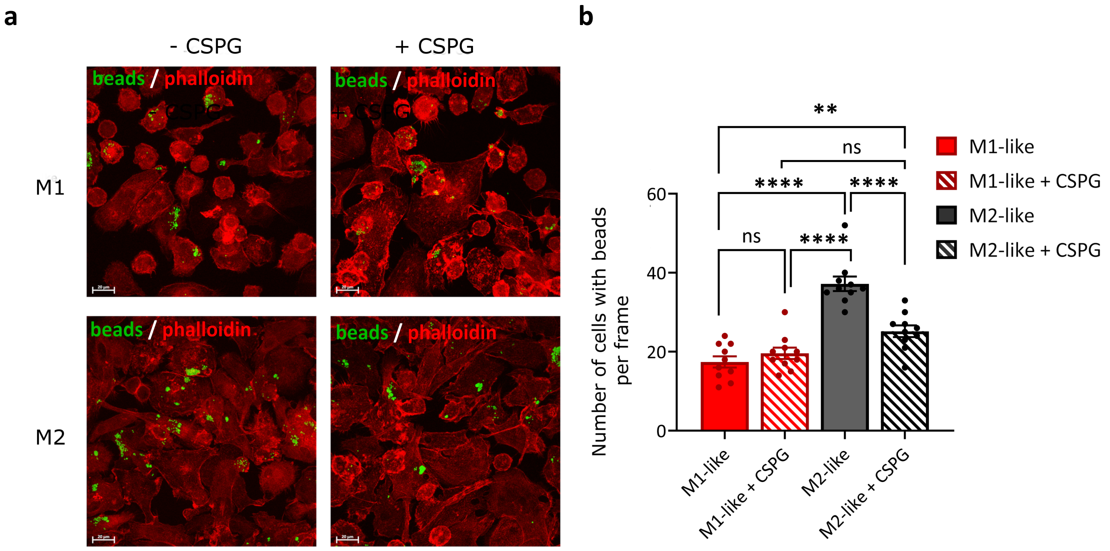

Supplementary Figure 8. CSPGs reduce phagocytosis exclusively in M2-like BMDMs. (a) ICC of green fluorescent latex beads phagocytosed by M1-like and M2-like BMDMs stained with Phallodin-568 (red). (b) Bar graphs showing number of cells with phagocytosed beads per frame. CSPG treatment reduces phagocytosis function only in M2-like BMDM. * $p < 0.05$, ** $p < 0.01$, *** $p < 0.001$, **** $p < 0.0001$ vs. control (no CSPGs). Results were assessed for normality using the Shapiro-Wilk test and one-way ANOVA with Tukey post hoc test was used to analyse differences between conditions. Data are shown as mean ± SEM (n = 10 per group; n = 5 randomly distributed frames per coverslip in a total of 2 coverslips per treatment).

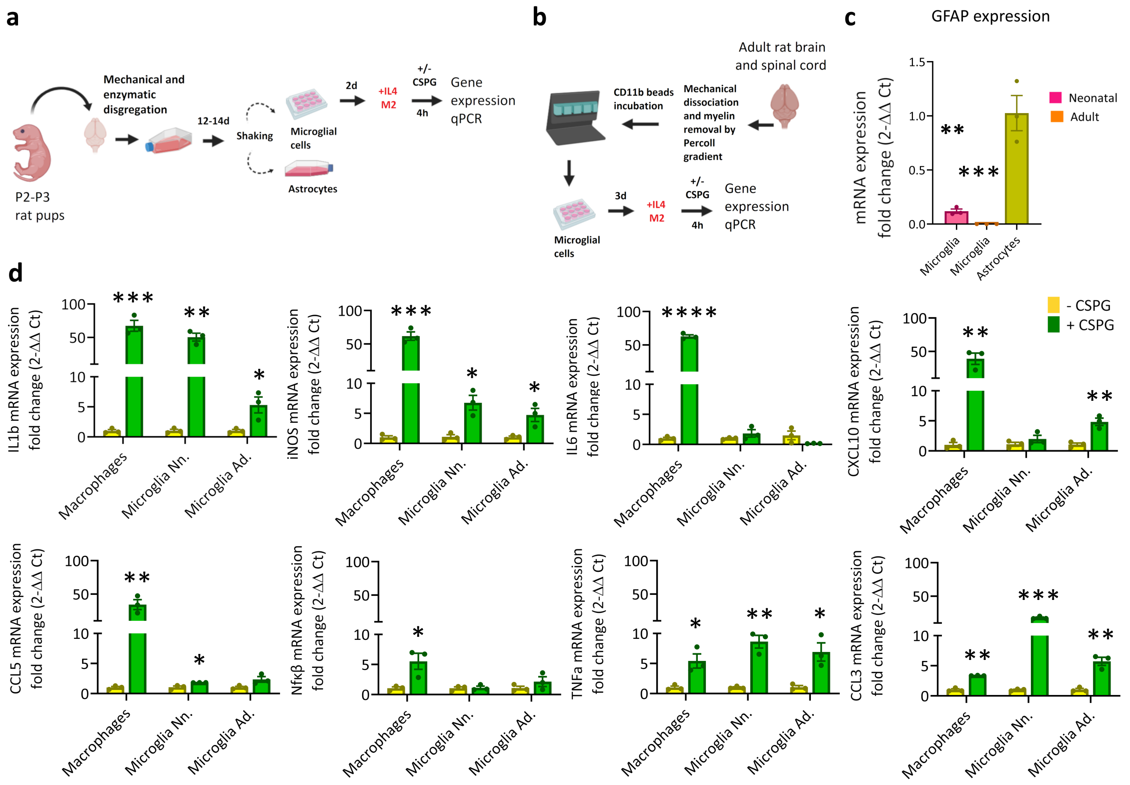

Supplementary Figure 9. CSPG immunomodulatory effects are more prominent in M2-like polarised macrophages than in M2-like polarised microglial cells. Experimental design of CSPG phenotype conversion studies in M2-like polarised (a) neonatal and (b) adult microglial cells in vitro. (c) mRNA level of GFAP determined by pPCR to evaluate astrocyte contamination after purification of microglial cells. mRNA levels were determined by qPCR. Results were assessed for normality using the Shapiro-Wilk test and one-way ANOVA with Tukey post hoc test was used to analyse differences between conditions. Data are shown as mean ± SEM (n = 3 per group). **p < 0.01, ***p < 0.001 vs. Astrocytes group. (d) Bar graphs showing genes significantly altered by CSPG treatment in M2 polarised BMDM macrophages compared to neonatal (Nn) and adult (Ad) microglial cells in vitro. mRNA levels were determined by qPCR. * p < 0.05, **p < 0.01, *** p < 0.001 vs. control (no CSPGs). Results were assessed for normality using the Shapiro-Wilk test and analysed using a two-tailed unpaired t test vs its own control. Data are shown as mean ± SEM (n = 3 per group).

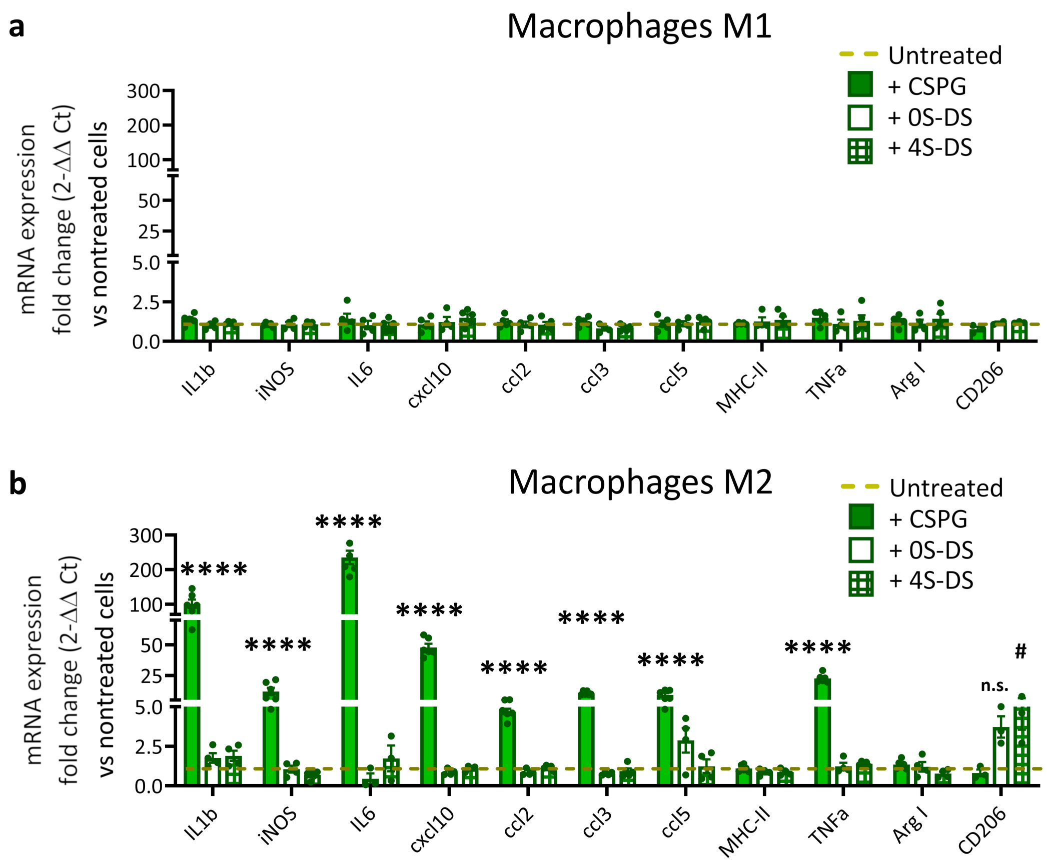

Supplementary Figure 10. CSPG digestion products do not influence pro-inflammatory gene expression in M1-like or M2-like BMDMs. Bar graphs showing the expression of inflammatory response genes in (a) M1-like BMDMs and (b) M2-like BMDMs after addition of glycosaminoglycan disaccharide digestion products, in comparison to CSPG stimulation. mRNA levels were determined by qPCR. Data were normalized with respect to control (no CSPGs). Results were assessed for normality using the Shapiro-Wilk test and one-way ANOVA with Tukey post hoc test was used to analyse differences between conditions. ****$p < 0.0001$ CSPG vs. control group (untreated); # $p < 0.05$ 4S-DS vs. control group. Data are presented as mean ± SEM (n = 14 per group). (M1-like BMDM n = 13 in control group, n = 5 in CSPG group, n = 4 in 0-DS group and n = 5 in 4-DS group; M2-like BMDM n = 14 in control group, n = 6 in CSPG group, n = 4 in 0-DS group and n = 5 in 4-DS group).

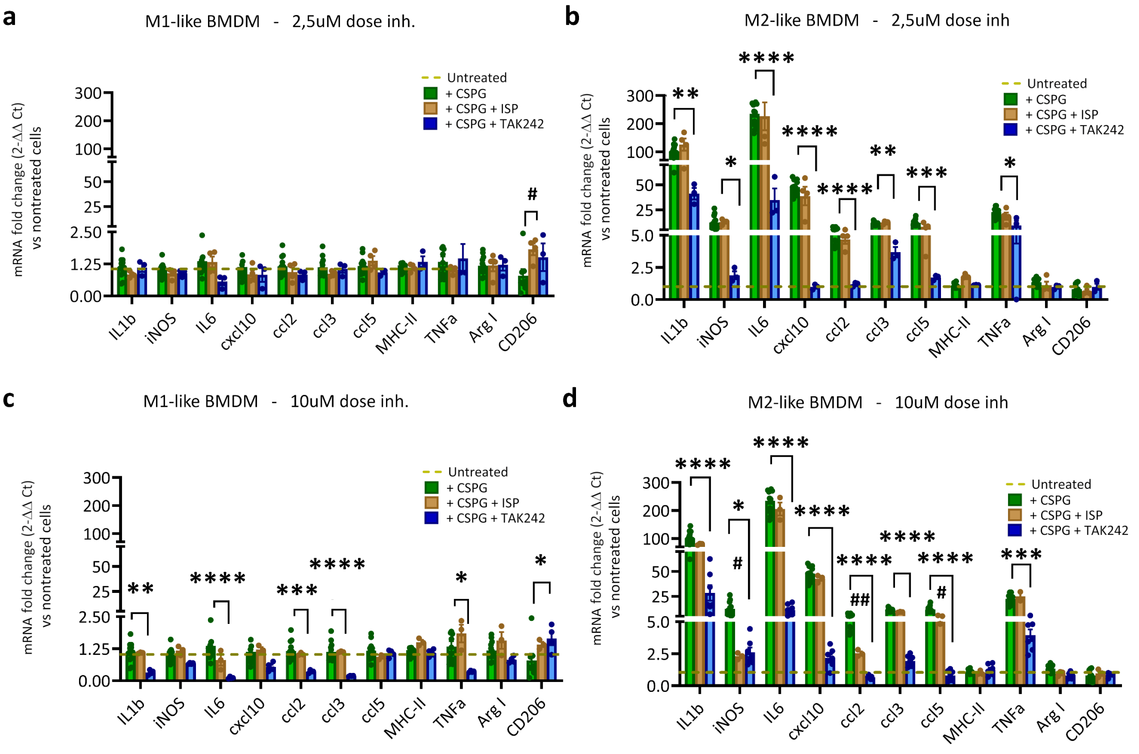

Supplementary Figure 11. CSPGs predominantly act via the TLR4 pathway, rather than via PTPσ, to cause M2-like polarized BMDMs to switch to a proinflammatory phenotype. Bar graphs showing the expression of inflammatory response genes in (a, c) M1 BMDMs and (b, d) M2 BMDMs comparing effect of two different concentrations (a-b) 2.5uM and (c-d) 10 uM of TLR4 inhibitor (TAK242) versus PTPσ inhibitor (ISP) under CSPG activation conditions. mRNA levels were determined by qPCR. Data were normalized with respect to control (no CSPGs). Results were assessed for normality using the Shapiro-Wilk test and one-way ANOVA with Tukey post hoc test was used to analyse differences between conditions. *p < 0.05, **p < 0.01, ***p < 0.001 ****p < 0.0001 CSPG+TAK242 vs. CSPG treated group; #p < 0.05, ##p < 0.01, CSPG+ISP vs. CSPG treated group. Data are presented as mean ± SEM (M1-like BMDM n = 13 in control group, n = 13 in CSPG group, n = 4 in CSPG + ISP 2,5 uM group, n = 3 in CSPG + ISP 10 uM group, , n = 3 in CSPG + TAK242 2,5 uM group and n = 4 in CSPG + TAK242 10 uM group; M2-like BMDM n = 13 in control group, n = 13 in CSPG group, n = 4 in CSPG + ISP 2,5 uM group, n = 3 in CSPG + ISP 10 uM group, , n = 3 in CSPG + TAK242 2,5 uM group and n = 7 in CSPG + TAK242 10 uM group).

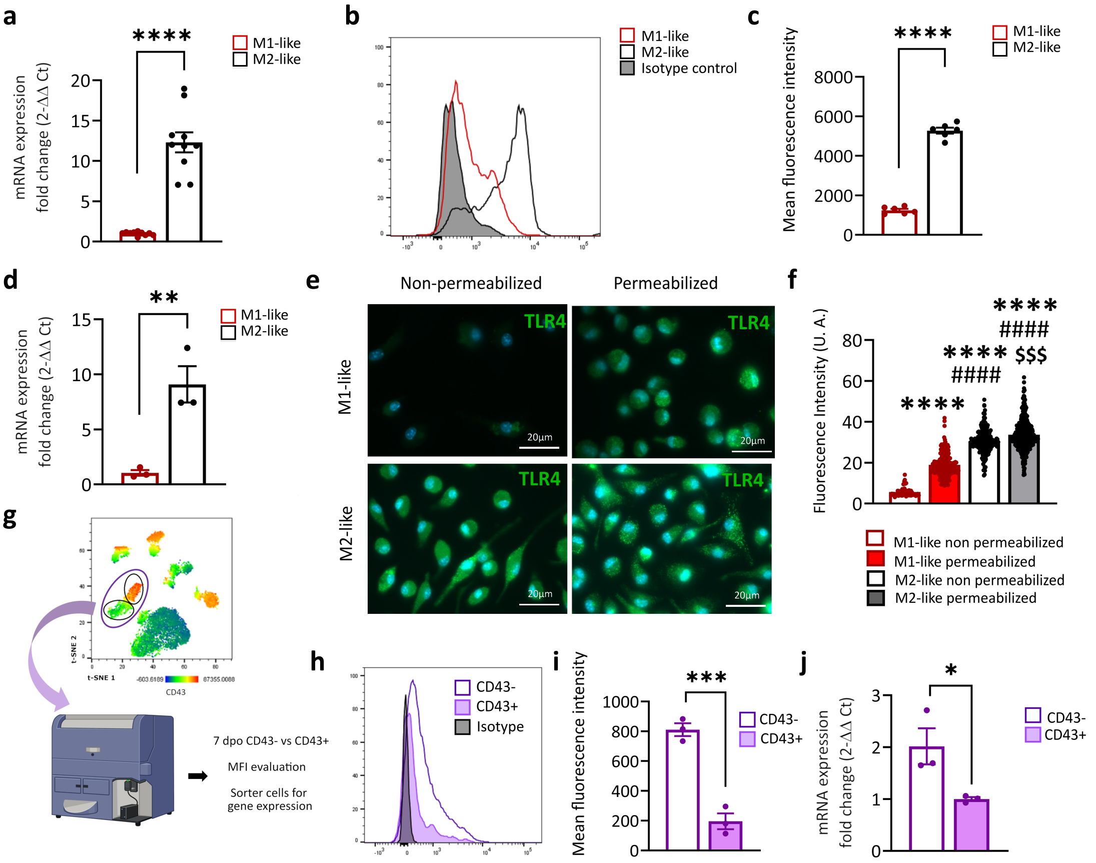
a
****
M1-like
M2-like
mRNA expression
fold change (2-ΔΔ Ct)
b
M1-like
M2-like
Isotype control
c
****
M1-like
M2-like
Mean fluorescence intensity
d
**
M1-like
M2-like
mRNA expression
fold change (2-ΔΔ Ct)
e
Non-permeabilized
Permeabilized
M1-like
M2-like
TLR4
20µm
f
****
####
$$$
Fluorescence Intensity (U. A.)
M1-like non permeabilized
M1-like permeabilized
M2-like non permeabilized
M2-like permeabilized
g
t-SNE 1
t-SNE 2
CD43
7 dpo CD43- vs CD43+
MFI evaluation
Sorter cells for
gene expression
h
CD43-
CD43+
Isotype
i
***
Mean fluorescence intensity
CD43-
CD43+
j
*
mRNA expression
fold change (2-ΔΔ Ct)
CD43-
CD43+

Supplementary Figure 12. The effect of CSPGs on immune cells is TLR4-expression dependent. M2-like polarized BMDM and CD43- monocytes/macrophages exhibit more TLR4 expression than M1-like and CD43+ respective counterparts. (a) Bar graph showing TLR4 gene expression in M1 and M2-like rat BMDM assessed by qPCR. Results were assessed for normality using the Shapiro-Wilk test and analysed using a two-tailed unpaired t test. ****$p < 0.0001$ M1-like vs. M2-like. Data are presented as mean ± SEM. (n = 10 per group; n = each BMDM polarized well). (b) FACS plot histogram of TLR4 expression in M1-like (red) and M2-like (black) BMDM. Grey colour represents the isotype control. (c) Bar graph showing protein TLR4 different expression between M1-like and M2-like BMDM assessed by flow cytometry. Result represented as mean fluorescence intensity shows higher TLR4 expression in M2-like compared with M1-like BMDM. Results were assessed for normality using the Shapiro-Wilk test and analysed using a two-tailed unpaired t test. ****$p < 0.0001$ M1-like vs. M2-like. Data are presented as mean ± SEM. (n = 6 per group; n = each BMDM polarized well). (d) Bar graph showing differential TLR4 gene expression in mouse M1- and M2-like BMDM assessed by qPCR. Results were assessed for normality using the Shapiro-Wilk test and analysed using a two-tailed unpaired t test. **$p < 0.01$ M1-like vs. M2-like. Data are presented as mean ± SEM. (n = 3 per group; n = each BMDM polarized well). (e) Immunocytochemistry of TLR4 (green) in M1-like M2-like BMDMs with or without permeabilization. Nuclei are labelled with DAPI (blue). (f) Bar graph showing TLR4 protein detection in M1-like and M2-like mouse BMDM with and without cell permeabilization. Results were assessed for normality using the Shapiro-Wilk test and one-way ANOVA with Tukey post hoc test was used to analyse differences between conditions. **** $p < 0.0001$ vs M1-like non permeabilized; ### $p < 0.001$ vs M1-like permeabilized; $$ $p < 0.01$ vs M2-like non permeabilized. Data are presented as mean ± SEM. (fluorescence measured in at least 50 cells at 3 different batches; n = 54 in M1-like non-permeabilized group, n = 179 in M1-like permeabilized group, n = 192 in M2-like non-permeabilized group and n = 397 in M2-like permeabilized group; n = each assessed cell). (g) Experimental design for cell sorting. (h) FACS plot histogram of TLR4 expression in CD43+ and CD43- monocytes/macrophages. Grey colour represents the isotype control. (i) Bar graph showing protein TLR4 different expression between in vivo CD43+ and CD43- monocyte/macrophages assessed by flow cytometry. Result represented as mean fluorescence intensity shows higher TLR4 expression in CD43- population compared with CD43+. (j) Bar graph showing differential TLR4 gene expression in rat sorted CD43- and CD43+ monocytes/macrophages assessed by qPCR. (i, j) Results were assessed for normality using the Shapiro-Wilk test and analysed using a two-tailed unpaired t test. *$p < 0.05$, **$p < 0.01$ and ***$p < 0.001$ CD43+ vs CD43-. Data are presented as mean ± SEM (n = 3 per group in CD43- vs CD43+).

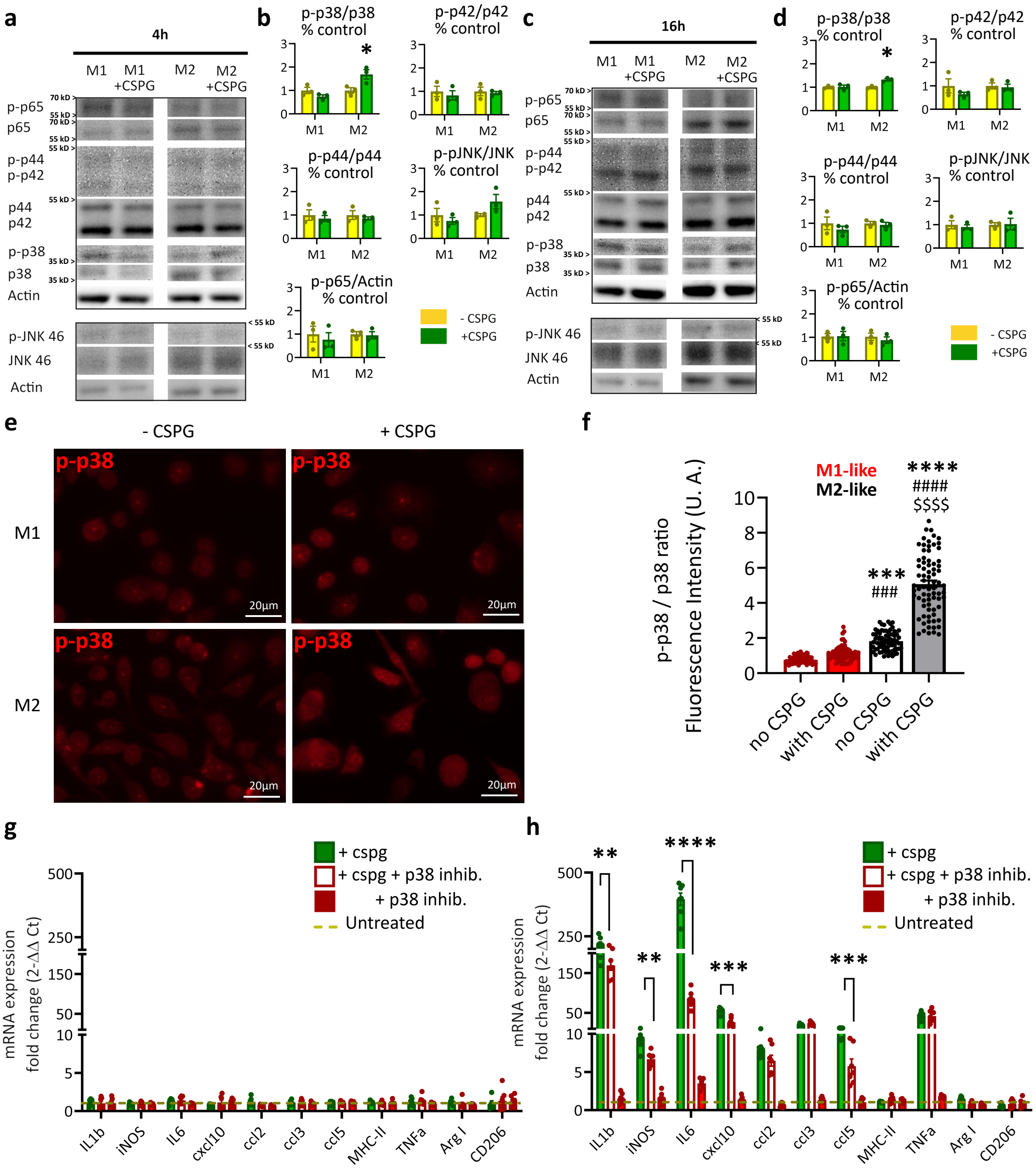
a
4h
M1
M1 +CSPG
M2
M2 +CSPG
p-p65
p65
p-p44
p-p42
p44
p42
p-p38
p38
Actin
p-JNK 46
JNK 46
Actin
b
p-p38/p38 % control
p-p42/p42 % control
p-p44/p44 % control
p-pJNK/JNK % control
p-p65/Actin % control
- CSPG
+CSPG
c
16h
d
e
- CSPG
+ CSPG
p-p38
20µm
f
M1-like
M2-like
p-p38 / p38 ratio
Fluorescence Intensity (U. A.)
no CSPG
with CSPG
g
h
+ cspg
+ cspg + p38 inhib.
+ p38 inhib.
Untreated
mRNA expression
fold change (2-ΔΔ Ct)
IL1b
iNOS
IL6
cxcl10
ccl2
ccl3
ccl5
MHC-II
TNFa
Arg I
CD206

Supplementary Figure 13. MAPK p38 pathway is affected by CSPG treatment in M2-like polarised BMDMs at 4 h and 16 h. (a) Representative Western blots and (b) quantitative analysis showing IL1b expression and inflammatory intracellular pathway activation in BMDMs after 4 h CSPG treatment. (c) Representative Western blots and (d) quantification analysis showing IL1b expression and inflammatory intracellular pathway activation in BMDMs after 16 h CSPG treatment. CSPG treatment enhanced the activation of p38 signalling in M2 BMDMs at both 4 and 16 h. (b, d) *$p<0.05$, **$p < 0.01$. Data are shown as mean ± SEM (n = 3 per group) and two-tailed unpaired t test was used to analyse differences between treatment conditions. (e) Immunocytochemistry of p-p38 (red) in M1-like M2-like BMDMs with or without CSPG. (f) Bar graph showing p-p38 protein detection in M1-like and M2-like mouse BMDM with and without CSPG treatment. CSPG activated p38 MAPK pathway in mouse M2-like BMDM. Results were assessed for normality using the Shapiro-Wilk test and one-way ANOVA with Tukey post hoc test was used to analyse differences between conditions. *** $p < 0.001$, **** $p < 0.0001$ vs M1-like without CSPG; ### $p < 0.001$ vs M1-like with CSPG; $$$$ $p < 0.0001$ vs M2-like without CSPG (fluorescence measured in at least 70 cells; n = 85 in M1-like + no CSPG group, n = 71 in M1-like + CSPG group, n = 72 in M2-like + no CSPG group and n = 75 in M2-like + CSPG group; n = each assessed cell). Data are presented as mean ± SEM. (g, h) MAPK p38 inhibition assay in M1-like and M2-like BMDM with CSPG stimulation. Bar graphs showing the expression of inflammatory response genes in (g) M1 BMDMs and (h) M2 BMDMs. mRNA levels were determined by qPCR. Data were normalized with respect to control (untreated - no CSPGs). Results were assessed for normality using the Shapiro-Wilk test and one-way ANOVA with Tukey post hoc test was used to analyse differences between conditions. *$p < 0.05$, **$p < 0.01$, ***$p < 0.001$ vs. CSPG treated group. Data are presented as mean ± SEM (n = 7 per group).

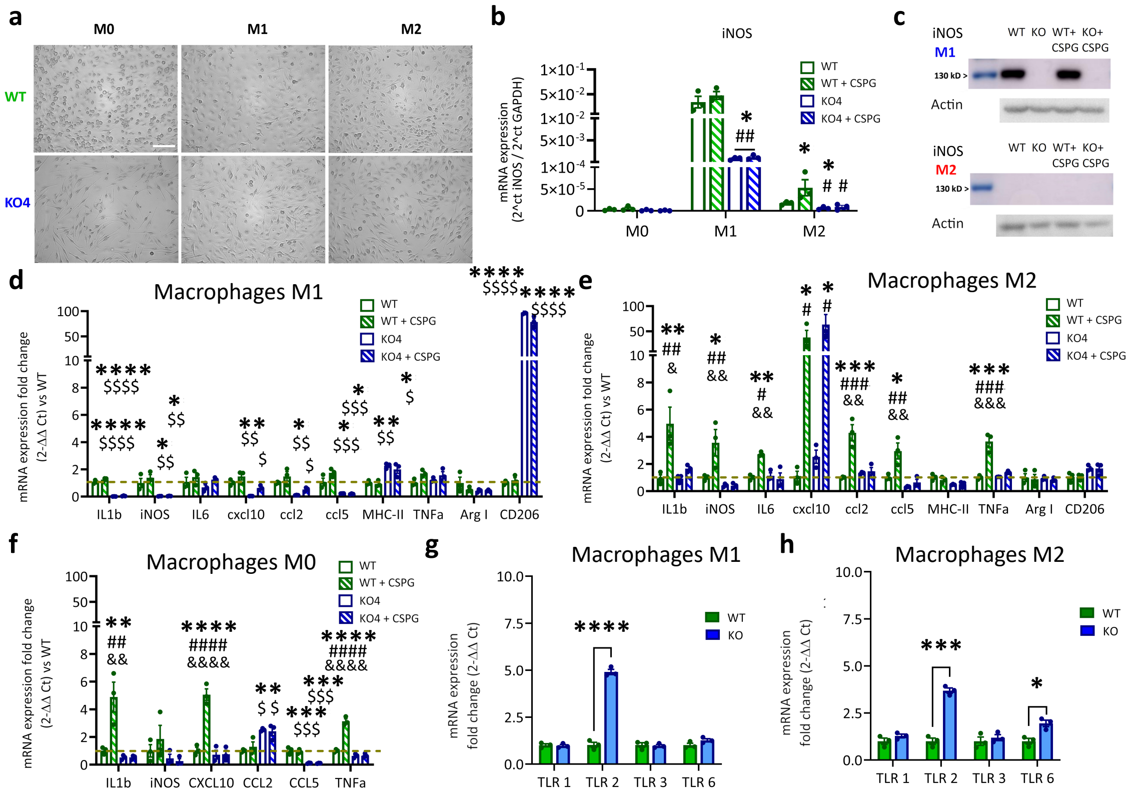
a
M0
M1
M2
WT
KO4
b
iNOS
mRNA expression (2^ct iNOS / 2^ct GAPDH)
WT
WT + CSPG
KO4
KO4 + CSPG
M0
M1
M2
c
iNOS
M1
WT KO WT+ CSPG KO+ CSPG
130 kD >
Actin
M2
d
Macrophages M1
mRNA expression fold change (2-ΔΔ Ct) vs WT
IL1b iNOS IL6 cxcl10 ccl2 ccl5 MHC-II TNFa Arg I CD206
e
Macrophages M2
IL1b iNOS IL6 cxcl10 ccl2 ccl5 MHC-II TNFa Arg I CD206
f
Macrophages M0
IL1b iNOS CXCL10 CCL2 CCL5 TNFa
g
Macrophages M1
mRNA expression fold change (2-ΔΔ Ct)
WT
KO
TLR 1 TLR 2 TLR 3 TLR 6
h
Macrophages M2
TLR 1 TLR 2 TLR 3 TLR 6

Supplementary Figure 14. WT and TLR4 KO macrophage polarization and their responses to CSPG treatment. (a) Images of bright field microscopy of BMDMWT and BMDMTRL4-/- showing morphology changes after M1 or M2 polarization. Scale bar corresponds to 100 µm. (b) Bar graphs showing iNOS gene expression assessed by qPCR in non-polarized and M1 and M2 polarized BMDMWT and BMDMTRL4-/-. ***p < 0.001, ****p < 0.0001 vs. WT; ##p < 0.01, ###p < 0.001, ####p < 0.0001 vs WT+CSPG. Results were assessed for normality using the Shapiro-Wilk test and one-way ANOVA with Tukey post hoc test was used to analyse significant differences. Data are shown as mean ± SEM (n=3 per group). (c) Representative Western Blot of iNOS protein in M1 and M2 BMDMs showing iNOS expression only in WT M1 conditions. Experiment was performed once. (d-f) Bar graphs comparing immunomodulatory effects of CSPG treatment (4h at 5ug/ml) between WT and TLR4-/- BMDM. Differences in inflammatory gene expression by CSPGs treatment were assessed by qPCR in (d) M1-like, (e) M2-like and (f) non-polarized (M0) BMDMs. Results were assessed for normality using the Shapiro-Wilk test and one-way ANOVA with Tukey post hoc test was used to analyse differences between conditions. *p < 0.05, **p < 0.01, ***p < 0.001 vs WT; $p < 0.05, $$p < 0.01, $$$p < 0.001 $$$$p < 0.0001 vs WT+CSPG; #p < 0.05, ##p < 0.01, ###p < 0.001, ####p < 0.0001 vs TLR4-/-; and &p < 0.05, &&p < 0.01, &&&p < 0.001 &&&&p < 0.0001 significance vs TLR4-/- + CSPGs. Data are shown as mean ± SEM (n=3 per group). (g-h) Bar graphs showing TLR gene expression differences between WT and TLR4-/- in M1-like (g) and M2-like (h) polarized BMDMs. ***p < 0.001, ****p < 0.0001 vs. WT BMDM. Results were assessed for normality using the Shapiro-Wilk test and analysed using a two-tailed unpaired t test. Data are shown as mean ± SEM (n = 3 per group).

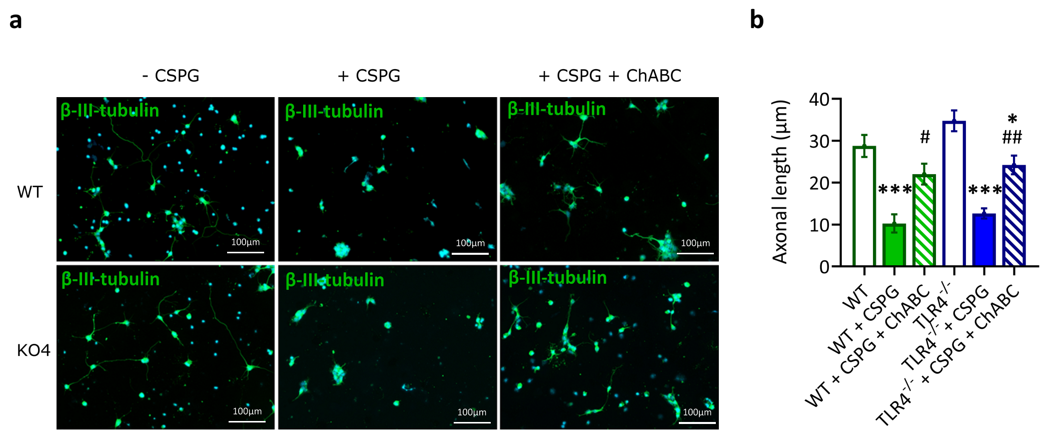

Supplementary Figure 15. Effect of CSPGs on neurite outgrowth is TLR4-independent. Axonal length assay. (a) Immunocytochemistry of β-III-tubulin (green) in WT and mouse TLR4-/- BMDMs under different conditions (untreated; +CSPG; +CSPG +ChABC) (Blue = DAPI). (b) Bar graph showing axonal length at different conditions. CSPGs promote axonal retraction in both WT and TLR4-/- neurons. This effect is partially rescued with CSPG digestion (ChABC). Results were assessed for normality using the Shapiro-Wilk test and one-way ANOVA with Tukey post hoc test was used to analyse differences between conditions. Data are shown as mean ± SEM (Between 80-210 neurons were analysed per condition, in triplicates: n = 162 in WT group, n = 82 in WT + CSPG group, n = 207 in WT + CSPG + ChABC group, n = 201 in TLR4-/- group, n = 171 in TLR4-/- + CSPG group and n = 165 in TLR4-/- + CSPG + ChABC group). ***p < 0.001 with vs without CSPG; #p < 0.05, ##p < 0.01 CSPG + ChABC group vs its own group with only CSPG.

Supplementary Table 1 – Flow cytometry panels

Flow cytometry - Panel 1				
**Antigen**	**Fluorochrome**	**Dilution**	**Pre-Conjugate**	**Company - Ref**
CD45	BUV395	1:250	-	BDBioscience-740258
CD11b	V450	1:300	-	BDBioscience-562108
CD43	PE-Cy7	1:150	-	Miltenyi Biotec - 130-107-721
CD45RA	APC	1:150	-	Miltenyi Biotec - 130-106-827
CD3	BV605	1:250	-	BDBioscience - 563949
CD4	PE	1:200	-	BDBioiscience - 551397
CD8	FITC	1:150	-	Miltenyi Biotec – 130-119-664
RP-1	BV786	1:200	-	BD Bioscience - 743058
Live/Dead	NIR (APC-Cy7)	1:1000	-	Biolegend - 423105
**Flow cytometry - Panel 2**				
**Antigen**	**Fluorochrome**	**Dilution**	**Pre-Conjugate**	**Company - Ref**
CD45	BUV395	1:250	-	BDBioscience-740258
CD11b	V450	1:300	-	BDBioscience-562108
CD43	PE-Cy7	1:150	-	Miltenyi Biotec - 130-107-721
HIS48	AF700*	1:200	* AF700 kit (ab269824)	ThermoFisher - 14-0570-82
CD45RA	BUV805	1:250	-	BDBioscience-741973
CD3	BV605	1:250	-	BDBioscience-563949
CD4	BUV737	1:250	-	BDBioscience-749058
CD8	BUV563	1:250	-	BDBioscience-748879
CD68	APC-Cy7	1:125	-	Miltenyi Biotec - 130-103-366
CD86	BV510	1:250	-	BDBioscience- 743212
INOS	AF647	1:300	-	Santa Cruz - sc-7271 AF647
MHC II	PerCP-Cy5.5	1:150	-	Miltenyi Biotec - 130-107-877
Arg I	PE-Cy5*	1:200	* PE-Cy5 kit (ab102893)	Santa Cruz - sc-271430
CD206	FITC	1:150	-	Bioss – bs-4727R-FITC
CD163	PE	1:300	-	GeneTex – GTX42934
Live/Dead	Yellow (BV570)	1:1000	-	

Supplementary Table 2 – TLR4 flow cytometry panel

Flow cytometry - TLR4 expression				
**Antigen**	**Fluorochrome**	**Dilution**	**Pre-Conjugate**	**Company - Ref**
CD45	BUV395	1:250	-	BDBioscience-740258
CD11b	V450	1:300	-	BDBioscience-562108
TLR4	AF647	1:300	-	Biotechne - NBP2-27149
Live/Dead	NIR (APC-Cy7)	1:1000	-	Biolegend - 423105

Supplementary Table 3 – Western blot antibodies used

Western blot antibodies		
**Antibody**	**Dilution**	**Company - Ref**
Rabbit anti rat p-JNK	1:1000	Cell Signalling – cs3033T
Mouse anti rat JNK	1:300	Santa Cruz – sc-7345
Rabbit anti rat p-ERK 1/2	1:1000	Cell Signalling – cs9106S
Mouse anti rat ERK 1/2	1:300	Santa Cruz – sc-514302
Rabbit anti rat p-p38	1:1000	Cell Signalling – cs4511T
Mouse anti rat p38	1:300	Santa Cruz – sc-7972
Rabbit anti rat p-p65	1:1000	Cell Signalling – cs3033T
Mouse anti rat p65	1:1000	Cell Signalling – cs6956T
Rabbit anti β-Actin	1:3000	Abcam – ab179467
Rabbit anti rat GAPDH	1:2000	Abcam – ab181602
Rabbit anti mouse iNOS	1:600	Abcam - ab15323
Mouse anti- β-Actin	1:5000	Sigma – A1978

Supplementary Table 4 – Immunochemistry primary antibodies list

Immunochemistry antibodies		
**Antibody**	**Dilution**	**Company - Ref**
mouse anti-CSPG	1:250	AbD Serotec
chicken anti-GFP	1:1000	Abcam - ab13970
Mouse anti- CS-56	1:300	Sigma - C8035
Mouse anti-GFAP-488	1:500	EMD Millipore - MAB3402X
Rabbit anti-CD206	1:300	Abcam – ab64693
Rabbit anti-iNOS	1:100	Abcam – ab15323
Mouse anti-NFH-488	1:500	EMD Millipore – MAB5256X
Mouse anti-TLR4	1:50	Santa Cruz - sc-293072
Mouse anti-β-III-tubulin	1:500	Neuromics – MO15013
Rabbit anti-p-p38	1:100	Cell Signalling – cs9211
Rabbit anti-P38	1:100	Cell Signalling – cs9212

Supplementary Table 5 – Gene expression analysis primers

Rat Primers					
**Actb1**	FW	GCAGGAGTACGATGAGTCCG	**IL17**	FW	GTTCAGTGTGTCCAAACGCC
	RW	ACGCAGCTCAGTAACAGTCC		RW	AGGGTGAAGTGGAACGGTTG
**AKT1**	FW	GCACTTTCCCCAGTTCTCCTAC	**IL18**	FW	TATCGACCGAACAGCCAACG
	RW	GCCCACAGTAGAAACATCCTC		RW	GATAGGGTCACAGCCAGTCC
**ArgI**	FW	ACATTGGCTTGCGAGACGTA	**Inos**	FW	GCCTAGTCAACTACAAGCCCC
	RW	ATCACCTTGCCAATCCCCAG		RW	AGAAACTTCCAGGGGCAAGC
**C1qa**	FW	GGCCGAGGGAAAAATGAGGA	**IRF3**	FW	TGGCTGCGAGTCTCAACTAC
	RW	GGGACGAGGTGTGGATTGAG		RW	GGTTTCGGGGTTCCCATGAT
**C3**	FW	ATCGAGGATGGTTCAGGGGA	**IRF7**	FW	GTGATGCTGGGGACCTCTTG
	RW	GCCTCTACCATGTCGCTACC		RW	GGTTGCGCTCAGTCATCAGA
**C3ar1**	FW	CTGTGAGCGGGACACCTTAG	**JAK2**	FW	GAGAAGGACCAGACTCCCCT
	RW	CATCTGGGATGGGGCTTGAA		RW	GCACGCACTTCGGTAAGAAC
**Caspase 1**	FW	GACCGAGTGGTTCCCTCAAG	**L-Sel**	FW	GGTACTAACAACCGCCACCA
	RW	GACGTGTACGAGTGGGTGTT		RW	CTCTAACGTGGGAGATGCCC
**CCL2**	FW	TAGCATCCACGTGCTGTCTC	**Map2K1**	FW	GAATGGATAGCCGACCTCCC
	RW	GAGCTTGGTGACAAATACTACAGC		RW	ACTCCACTGGGCAGTTTTGG
**CCL3**	FW	CGGGTGTCATTTTCCTGACCA	**MapK1**	FW	CATTGTCTCACTGTGTTGCCA
	RW	GGAGGTTTGGGGGTTCCTTG		RW	CCAGGAAAGTCAGAAGGCACT
**CCL5**	FW	CATATGGCTCGGACACCACT	**MapK3**	FW	CACTGGCTTTCTGACCGAGT
	RW	GACTGCAAGGTTGGAGCACT		RW	GCCCACAGACCAGATGTCAA
**CCR2**	FW	TAGGGCTGTGAGGCTCATCT	**MapK8**	FW	TGCTGGTGATAGATGCGTCC
	RW	GCTCCCCAGTAGAAGGGGTA		RW	CCAGACGTTGATGTACGGGT
**CD163**	FW	CTGAAATCCTCGGGTTGGCA	**MHCII**	FW	AGAGACCATCTGGAGACTTG
	RW	TGTAGCTGTGGTCATCCGTG		RW	CATCTGGGGTGTTGTTGGA
**CD206**	FW	GTGGAGTGATGGAACCCCAG	**MMP2**	FW	GGGTGGTGGTCACAGCTATT
	RW	CTGTCCGCCCAGTATCCATC		RW	CCCAGCCAGTCCGATTTGAT
**CD68**	FW	ACCCGGAGACGACAATCAAC	**MMP9**	FW	GCATCTGTATGGTCGTGGCT
	RW	CTTGGTGGCCTACAGAGTGG		RW	TGCAGTGGGACACATAGTGG
**CD86**	FW	AGACATGTGTAACCTGCACCAT	**MMP12**	FW	GAGAGCGAATTTGCTGAATGGT
	RW	ACCGACTTTTTCCGGTCCTG		RW	GGTGTCCAGTTGCCCAGTTA
**ChABC**	FW	AGAGCCGTAGGCGTCTCTCT	**MyD88**	FW	AACCCAGAACCGAGGACCTA

	RW	AGCGTTGAGGGTCATCTCTC		RW	TGGCTATGCGTGGTGAAGTG
**COX-2**	FW	GATGACGAGCGACTGTTCCA	**Nfkb1a**	FW	CTCAAGAAGGAGCGGTTGGT
	RW	TGGTAACCGCTCAGGTGTTG		RW	CCAAGTGCAGGAACGAGTCT
**CSF1**	FW	CACTAGCGAGCAAGGAAGCG	**Nlrp1a**	FW	GCCTGGGACGAACACATCAT
	RW	CCATCCATGTCGAAGAAGGGC		RW	TTCAAAGCAGGAAGCCAGTGA
**CSF2**	FW	CCAACCCCGGAAACTGACTG	**Nlrp3**	FW	TGCATGCCGTATCTGGTTGT
	RW	CTCCTCATTTCTGGACCGGC		RW	ATGTCCTGAGCCATGGAAGC
**CSF3**	FW	TCTGGCAGCAGATGGAAAGC	**PTPs**	FW	GCTACCGCGTCTACTACACC
	RW	CTGTCTCCAGGAAGCTCTGC		RW	TCACCATTTCCTGGGCATCC
**CX3CR1**	FW	CAGCTGCTCAGGACCTCAC	**RelA (p65)**	FW	TGTATTTCACGGGACCTGGC
	RW	CCAGACCGAACGTGAAGACA		RW	CAGGCTAGGGTCAGCGTATG
**CXCL2**	FW	GCTGTCCCTCAACGGAAGAA	**Rorc**	FW	AACATCTCGGGAGTTGCTGG
	RW	CAGGTACGATCCAGGCTTCC		RW	AGGAGTAGGCCACATTGCAC
**CXCL5**	FW	CGGAATGCACTTGCACTGGT	**S100A10**	FW	CACACCTTGATGCGTCCTCT
	RW	GAACCAGCCCTTCTTTCTTGC		RW	GGCAACCGGATGCAAACAAT
**CXCL10**	FW	CCGCATGTTGAGATCATTGCC	**Serping1**	FW	AGGCTAACTGGCTTCGTAGG
	RW	CTCTCTGCTGTCCATCGGTC		RW	CGGGAGCCATCTCTTTCAGG
**CXCR2**	FW	CATCGTAGAGCTACAGCAGGATT	**Smad1**	FW	TCAATAGAGGAGATGTTCAAGCAGT
	RW	CTGACAGAGTAAAGGGCGGG		RW	GAAACCATCCACCAACACGC
**FcRLs**	FW	AGTTATAAGGTGCACGGGGC	**Smad3**	FW	CTGGGCAAGTTCTCCAGAGTT
	RW	GTGTAGCTCGAAGAACCGCT		RW	GAAGGGCAGGATGGACGAC
**FoxP3**	FW	GTCTCCAGTACCCCCAAATTC	**STAT1**	FW	GGAAGGGGCCATCACATTCA
	RW	GCTGAAGACGTGTGCATCCTAT		RW	CTGGAGACATGGGAAGCAGG
**IFNg**	FW	ATCCATGAGTGCTACACGCC	**STAT3**	FW	CCTGAAGCTGACCCAGGTG
	RW	TCGTGTTACCGTCCTTTTGC		RW	TCCATGTCAAACGTGAGCGA
**GAPDH**	FW	AGTGCCAGCCTCGTCTCATA	**STAT4**	FW	TGGCTGAAAACATCCCCGAA
	RW	GGTAACCAGGCGTCCGATAC		RW	CCCGTTCTGTCGGTCTTGAA
**Gata-3**	FW	GGACCGGCTGGGAATTACAC	**STAT5a**	FW	CTTGTCCTGAACGCTGGACT
	RW	GCCGGATGCAAAGAACACTC		RW	CTACTTTAGCCAGACCCCGC
**GPR34**	FW	ATGACACTCAAGTGCAGGGC	**STAT6**	FW	AGCCGGAACAGATGGGAAAG
	RW	CCCCGTTTGGAGCCAAGTAG		RW	CTCTGGAGTAGGAAGGGGCT
**IL1b**	FW	CAGCTTTCGACAGTGAGGAGA	**T-bet (Tbx21)**	FW	AAGGCAGTATGCCAGGGAAC
	RW	TTGTCGAGATGCTGCTGTGA		RW	TTGGAAGCCCCCTTGTTGTT

**IL4**	FW	GTACCGGGAACGGTATCCAC	**Ticam1 (TRIF)**	FW	GATGCCGAAAAGGACCCATC
	RW	ACATCTCGGTGCATGGAGTC		RW	GGTCATGGATGGGGGAGGAT
**IL6**	FW	TTTCTCTCCGCAAGAGACTTCC	**TGFb**	FW	CTGCTGACCCCCACTGATAC
	RW	TGTGGGTGGTATCCTCTGTGA		RW	AGCCCTGTATTCCGTCTCCT
**IL10**	FW	CCTCTGGATACAGCTGCGAC	**TLR4**	FW	GAGGACAATGCTCTGGGGAG
	RW	GTAGATGCCGGGTGGTTCAA		RW	ATGGGTTTTAGGCGCAGAGT
**IL13**	FW	ATGGTATGGAGCGTGGACCT	**TNFa**	FW	ATGGGCTCCCTCTCATCAGT
	RW	ACTGGAGATGTTGGTCAGGG		RW	GCTTGGTGGTTTGCTACGAC
**Mouse Primers**					
**GAPDH**	FW	CGGTGCTGAGTATGTCGTGGAGT	**CXCL10**	FW	ATGACGGGCCAGTGAGAATG
	RW	CGTGGTTCACACCCATCACAAA		RW	TCAACACGTGGGCAGGATAG
**CCL2**	FW	TGCCCTAAGGTCTTCAGCAC	**IL1b**	FW	ACAGAATATCAACCAACA AGTGATATTCTC
	RW	AAGGCATCACAGTCCGAGTC		RW	GATTCTTTCCTTTGAGGCCCA
**CCL3**	FW	TGCCAAGTAGCCACATCGAG	**IL6**	FW	CTGGGGATGTCTGTAGCTCA
	RW	GAGATGGGGGTTGAGGAACG		RW	CTGTGAAGTTCTCTCTCCGG
**CCL5**	FW	TGCTCCAATCTTGCAGTCGT	**TNFa**	FW	TTCTATGGCCCAGACCCTCA
	RW	GCAAGCAATGACAGGGAAGC		RW	GTTTGCTACGACGTGGGCTA
**MHC-II**	FW	CAAGATCAAAGTGCGCTGGT	**CD206**	FW	TGGATGGATGGGAGCAAAGT
	RW	GCTCAACATCTTGCTCCAGG		RW	GCTGCTGTTATGTCTCTGGC
**iNOS**	FW	TGGCTCGCTTTGCCACGGAC	**Arg I**	FW	CTGAGCTTTGATGTCGACGG
	RW	GCTGCGACAGCAGGAAGGCA		RW	TCCTCTGCTGTCTTCCCAAG

Supplementary table 6 – Baseline Ct counts between WT vs KO4 BMDM

	Mouse BMDM baseline gene expression w/o CSPG									
	Ct gene of interest - Ct GAPDH									
	M1					M2				
	WT		KO4		WT vs KO4	WT		KO4		WT vs KO4
	Mean	SEM	Mean	SEM		Mean	SEM	Mean	SEM	
**IL1b**	**5,780**	0,303	**12,447**	0,260	****	**12,648**	1,008	**12,808**	0,504	n.s.
**iNOS**	**5,055**	0,860	**10,223**	0,576	*	**15,547**	0,708	**17,758**	0,960	*
**IL6**	**13,777**	0,597	**14,593**	0,986	n.s.	**14,557**	0,503	**14,363**	0,658	n.s.
**CXCL10**	**5,650**	0,486	**10,253**	0,206	**	**13,083**	0,670	**12,697**	0,488	n.s.
**CCL2**	**5,983**	0,159	**9,293**	0,178	*	**9,220**	0,100	**8,808**	0,087	n.s.
**CCL5**	**0,110**	0,113	**1,468**	0,036	*	**10,332**	0,147	**11,407**	0,356	n.s.
**MHC-II**	**3,553**	0,224	**2,378**	0,129	**	**4,992**	0,223	**5,640**	0,238	*
**TNFa**	**6,637**	0,339	**6,483**	0,313	n.s.	**6,993**	0,223	**6,925**	0,013	n.s.
**Arg I**	**12,210**	1,428	**12,140**	0,428	n.s.	**5,972**	0,701	**5,958**	0,218	n.s.
**CD206**	**12,425**	0,215	**5,530**	0,308	****	**2,822**	0,568	**2,160**	0,265	n.s.

*p < 0.05 **p < 0.01 ****p < 0.001 versus WT group. Results were assessed for normality using the Shapiro-Wilk test and analysed using a two-tailed unpaired t test. Data are shown as mean ± SEM

Supplementary Table 7 – Cell lines used

Cell lines used		
**Source**	**Authentication**	**Others**
HEK293T cells: ATCCATCC Sales Order: SO0591466. FTA Barcode: STRB2921	HEK293T cells: ATCC Cell line authentication	Mycoplasma contamination: not tested. Commonly misidentified lines: none used.
**Methods**		
Seventeen short tandem repeat (STR) loci plus the gender determining locus, Amelogenin, were amplified using the commercially available PowerPlex® 18D Kit from Promega. The cell line sample was processed using the ABI Prism® 3500xl Genetic Analyzer. Data were analyzed using GeneMapper® ID-X v1.2 software (Applied Biosystems). Appropriate positive and negative controls were run and confirmed for each sample submitted. Data Interpretation: Cell lines were authenticated using Short Tandem Repeat (STR) analysis.		

Supplementary Table 8 – Detailed statistics

Figures p-values						
**Figure**	**Graph**	**Analysis**	**Groups compared**	**Comparison**	**p-value**	**Significance**
**Figure 1.**	d	Unpaired *t*-test (two-tailed)	LV-ChABC vs LV-GFP	7d	0.0127	*
	e	Unpaired *t*-test (two-tailed)	LV-ChABC vs LV-GFP	7d	0.0492	*
	j	Two-way ANOVA	LV-ChABC vs LV-GFP	7d	0.019	*
	k	Two-way ANOVA	LV-ChABC vs LV-GFP	7d	0.004	**
	m	Unpaired *t*-test (two-tailed)	LV-ChABC vs LV-GFP	7d	0.005 in CD43low and 0.048 in CD43high	** *
**Figure 2.**	d	Two-way ANOVA	Naïve vs both treated groups	6h	<0.0001	****
				12h	<0.0001	****
			Naïve vs LV-GFP	1d	0.0015	**
			Naïve vs LV-ChABC	1d	0.01	*
			Naïve vs LV-GFP	3d	0.05	*
			Naïve vs LV-ChABC	3d	0.0038	**
			Naïve vs LV-GFP	7d	0.0073	**
			LV-GFP vs LV-ChABC	7d	0.0179	#
	g	Unpaired *t*-test (two-tailed)	LV-GFP vs LV-ChABC	Dimension 1	0.0033	**
	j	Unpaired *t*-test (two-tailed)	LV-GFP vs LV-ChABC	CCR2	0.0098	**
				MHC-II	0.0097	**
				L-Sel	0.0074	**
				CCL2	0.01396	*
				iNOS	0.035	*
				Smad3	0.0079	**
				CXCL10	0.050	*
				CXCR2	0.050	*
				MMP12	0.0476	*
				IL1b	0.0023	**
				TNFa	0.035	*
				CD68	0.0184	*
				CCL5	0.0376	*
				IL18	0.028	*
				MMP9	0.040	*
				CSF1	0.037	*
				TLR4	0.05	*
				C1qa	0.035	*

				Nlrp1a	0.049	*
**Figure 3.**	a	Unpaired *t*-test (two-tailed)	LV-GFP vs LV-ChABC	MHC-II	0.037	*
	c	Unpaired *t*-test (two-tailed)	LV-GFP vs LV-ChABC	iNOS	0.004	**
				CD68	0.003	**
				MHC-II	0.034	*
	e	Unpaired *t*-test (two-tailed)	LV-GFP vs LV-ChABC	CD86	0.011	*
				MHC-II	0.033	*
	g	Unpaired *t*-test (two-tailed)	LV-GFP vs LV-ChABC	iNOS	0.009	**
				CD68	0.007	**
				MHC-II	0.035	*
	j	Unpaired *t*-test (two-tailed)	Macrophages vs microglia	Gpr34	0.00013	***
				FcRls	0.0007	***
				CCR2	<0.0001	****
	k	Unpaired *t*-test (two-tailed)	Macrophages LV-GFP LV-ChABC	iNOS	0.021	*
				MHC-II	0.006	**
				CD68	0.033	*
			Microglia LV-GFP LV-ChABC	MHC-II	0.047	*
				Arg I	0.028	*
**Figure 4.**	g	Unpaired *t*-test (two-tailed)	LV-GFP vs LV-ChABC	CS-56 – all area	0.0011	**
	h	Unpaired *t*-test (two-tailed)	LV-GFP vs LV-ChABC	CS56 - perilesional	0.03	*
	i	Unpaired *t*-test (two-tailed)	LV-GFP vs LV-ChABC	CD206	0.048	*
	j	Unpaired *t*-test (two-tailed)	LV-GFP vs LV-ChABC	iNOS	0.025	*
**Figure 5.**	b	Unpaired *t*-test (two-tailed)	LV-GFP vs LV-ChABC	TCD4+	0.027	*
				TCD8+	0.004	**
	e	Unpaired *t*-test (two-tailed)	LV-GFP vs LV-ChABC	TCD4+ TNFa	0.024	*
				TCD4+ Tbet	0.027	*
**Figure 6.**	d	Unpaired *t*-test (two-tailed)	M1-like vs M1-like + CSPG	CCL3	0.018	*
	e	Unpaired *t*-test (two-tailed)	M2-like vs M2-like + CSPG	ÌL1b	0.0005	***
				iNOS	0.0005	***
				IL6	<0.0001	****
				CXCL10	0.01	**
				CCL5	0.01	**
				TNFa	0.0011	**
				CCL3	0.002	**
	g			iNOS	0.0017	**

		Unpaired *t*-test (two-tailed)	M1-like vs M1-like + CSPG	IL1b	0.021	*
	h	Unpaired *t*-test (two-tailed)	M2-like vs M2-like + CSPG	CCL5	0.002	***
				iNOS	0.009	**
				IL1b	0.016	*
				IL6	0.016	*
				MHC-II	0.0001	***
				C1q1	0.0015	**
				ArgI	0.038	*
**Figure 7.**	b	One-way ANOVA	1e8 GC/ml vs 0 GC/ml	ChABC	0.0001	***
			1e8 GC/ml vs 2e7 GC/ml	ChABC	0.0001	###
			1e8 GC/ml vs 4e7 GC/ml	ChABC	0.0002	&&&
	e	One-way ANOVA	CSPG vs untreated	M2 – IL1b	<0.0001	****
			CSPG vs CSPG+ChABC	M2 – IL1b	0.0004	###
			CSPG vs ChABC	M2 – IL1b	<0.0001	&&&&
			CSPG+ChABC vs untreated	M2 – IL1b	0.002	**
			CSPG+ChABC vs ChABC	M2 – IL1b	0.0021	&&
		One-way ANOVA	CSPG vs untreated	M2 – IL6	<0.0001	****
			CSPG vs CSPG+ChABC	M2 – IL6	<0.0001	####
			CSPG vs ChABC	M2 – IL6	<0.0001	&&&&
		One-way ANOVA	CSPG vs untreated	M2 – CCL5	<0.0001	****
			CSPG vs CSPG+ChABC	M2 – CCL5	<0.0001	####
			CSPG vs ChABC	M2 – CCL5	<0.0001	&&&&
		One-way ANOVA	CSPG vs untreated	M2 – CXCL10	0.0002	***
			CSPG vs CSPG+ChABC	M2 – CXCL10	0.0013	##
			CSPG vs ChABC	M2 – CXCL10	0.0003	&&&
	f	One-way ANOVA	CSPG+ChABC vs untreated	M1 – IL1b	0.0001	***
			CSPG+ChABC vs CSPG	M1 – IL1b	0.0006	$$$
			CSPG+ChABC vs ChABC	M1 – IL1b	0.009	&&
		One-way ANOVA	CSPG+ChABC vs untreated	M1 – IL6	<0.0001	****
			CSPG+ChABC vs CSPG	M1 – IL6	0.0013	$$
			ChABC vs untreated	M1 – IL6	0.0006	***
			ChABC vs CSPG	M1 – IL6	0.018	$
		One-way ANOVA	CSPG+ChABC vs untreated	M1 – CCL5	<0.0001	****
			CSPG+ChABC vs CSPG	M1 – CCL5	<0.0001	$$$$
			ChABC vs untreated	M1 – CCL5	<0.0001	****
			ChABC vs CSPG	M1 – CCL5	<0.0001	$$$$
		One-way ANOVA	CSPG+ChABC vs untreated	M1 – CXCL10	0.0029	**
			CSPG+ChABC vs CSPG	M1 – CXCL10	0.0012	$$
			CSPG+ChABC vs ChABC	M1 – CXCL10	0.002	&&
	h	Unpaired *t*-test (two-tailed)	Macrophages LV-GFP LV-ChABC	IL1b	0.0007	***
				CXLC10	0.015	*
				IL1b	0.017	*

			Microglia LV-GFP LV-ChABC	CCL5	0.043	*
**Figure 8.**	b	One-way ANOVA	CSPG + TAK242 vs control	M1 – IL1b	0.0001	***
			TAK242 vs control	M1 – IL1b	0.0089	**
			CSPG + TAK242 vs CSPG	M1 – IL1b	0.0001	&&&
			TAK242 vs CSPG	M1 – IL1b	0.0007	&&&
		One-way ANOVA	CSPG + TAK242 vs control	M1 – iNOS	0.0001	***
			TAK242 vs control	M1 – iNOS	0.0029	**
			CSPG + TAK242 vs CSPG	M1 – iNOS	0.0001	&&&
			TAK242 vs CSPG	M1 – iNOS	0.0015	&&
		One-way ANOVA	CSPG + TAK242 vs control	M1 – IL6	0.0003	***
			TAK242 vs control	M1 – IL6	0.0009	***
			CSPG + TAK242 vs CSPG	M1 – IL6	0.0001	&&&
			TAK242 vs CSPG	M1 – IL6	0.0001	&&&
		One-way ANOVA	CSPG + TAK242 vs control	M1 – CCL2	0.001	**
			TAK242 vs control	M1 – CCL2	0.0073	**
			CSPG + TAK242 vs CSPG	M1 – CCL2	0.0001	&&&
			TAK242 vs CSPG	M1 – CCL2	0.0001	&&&
		One-way ANOVA	CSPG + TAK242 vs control	M1 – CCL3	0.0001	***
			TAK242 vs control	M1 – CCL3	0.0001	***
			CSPG + TAK242 vs CSPG	M1 – CCL3	0.0001	&&&
			TAK242 vs CSPG	M1 – CCL3	0.0001	&&&
		One-way ANOVA	CSPG + TAK242 vs control	M1 – TNFa	0.0001	***
			TAK242 vs control	M1 – TNFa	0.0001	***
			CSPG + TAK242 vs CSPG	M1 – TNFa	0.0001	&&&
			TAK242 vs CSPG	M1 – TNFa	0.0001	&&&
		One-way ANOVA	TAK242 vs CSPG	M1 – CD206	0.031	&
			TAK242 vs CSPG	M1 – CD206	0.014	&
			TAK242 vs control	M1 – CD206	0.028	*
	c	One-way ANOVA	CSPG vs control	M2 – IL1b	<0.0001	****
			CSPG vs CSPG + TAK242	M2 – IL1b	<0.0001	####
			CSPG vs TAK242	M2 – IL1b	<0.0001	$$$$
		One-way ANOVA	CSPG vs control	M2 – iNOS	<0.0001	****
			CSPG vs CSPG + TAK242	M2 – iNOS	<0.0001	####
			CSPG vs TAK242	M2 – iNOS	<0.0001	$$$$
		One-way ANOVA	CSPG vs control	M2 – IL6	<0.0001	****
			CSPG vs CSPG + TAK242	M2 – IL6	<0.0001	####
			CSPG vs TAK242	M2 – IL6	<0.0001	$$$$

		One-way ANOVA	CSPG vs control	M2 – CXL10	<0.0001	****
			CSPG vs CSPG + TAK242	M2 – CXL10	<0.0001	####
			CSPG vs TAK242	M2 – CXL10	<0.0001	$$$$
		One-way ANOVA	CSPG vs control	M2 – CCL2	<0.0001	****
			CSPG vs CSPG + TAK242	M2 – CCL2	<0.0001	####
			CSPG vs TAK242	M2 – CCL2	<0.0001	$$$$
			CSPG + TAK242 vs control	M2 – CCL2	0.042	*
		One-way ANOVA	CSPG vs control	M2 – CCL3	<0.0001	****
			CSPG vs CSPG + TAK242	M2 – CCL3	<0.0001	####
			CSPG vs TAK242	M2 – CCL3	<0.0001	$$$$
		One-way ANOVA	CSPG vs control	M2 – CCL5	<0.0001	****
			CSPG vs CSPG + TAK242	M2 – CCL5	<0.0001	####
			CSPG vs TAK242	M2 – CCL5	<0.0001	$$$$
		One-way ANOVA	CSPG vs control	M2 – TNFa	<0.0001	****
			CSPG vs CSPG + TAK242	M2 – TNFa	<0.0001	####
			CSPG vs TAK242	M2 – TNFa	<0.0001	$$$$
	f	Unpaired *t*-test (two-tailed)	KO4 vs KO4 + CSPG	M1 – IL1b	0.05	*
		Unpaired *t*-test (two-tailed)	KO4 vs KO4 + CSPG	M1 – CXCL10	0.004	**
		Unpaired *t*-test (two-tailed)	KO4 vs KO4 + CSPG	M1 – CCL2	0.008	**
	g	Unpaired *t*-test (two-tailed)	WT vs WT + CSPG	M2 – IL1b	0.031	*
		Unpaired *t*-test (two-tailed)	WT vs WT + CSPG	M2 – iNOS	0.05	*
		Unpaired *t*-test (two-tailed)	WT vs WT + CSPG	M2 – IL6	0.0003	***
		Unpaired *t*-test (two-tailed)	WT vs WT + CSPG	M2 – CXCL10	0.05	*
		Unpaired *t*-test (two-tailed)	WT vs WT + CSPG	M2 – CCL2	0.0053	**
		Unpaired *t*-test (two-tailed)	WT vs WT + CSPG	M2 – CCL5	0.032	*
		Unpaired *t*-test (two-tailed)	WT vs WT + CSPG	M2 - TNFa	0.0033	**
	h	Unpaired *t*-test (two-tailed)	KO4 vs KO4 + CSPG	M2 – CXCL10	0.036	*

Supplementary Figures. p-values						
**Figure**	**Graph**		**Groups compared**	**Comparison**	**p-value**	
**Extended data Fig 1.**	b	One-way ANOVA	GFP vs 6hpo	ChABC	<0.0001	****
			GFP vs 12hpo	ChABC	<0.0001	****
			GFP vs 1dpo	ChABC	<0.0001	****
			GFP vs 3dpo	ChABC	0.0038	***
			GFP vs 7dpo	ChABC	<0.0001	****
			GFP vs 14dpo	ChABC	<0.0001	****
	d	Two-way ANOVA	LV-GFP vs LV-ChABC	7d	0.0296	*
**Extended data Fig 3.**	a	Two-way ANOVA	Naïve vs both treatments	Dim 1 – 6h	<0.0001	****
			Naïve vs both treatments	Dim 1 – 12h	<0.0001	****
			Naïve vs both treatments	Dim 1 – 1d	<0.0001	****
			LV-GFP vs Naïve	Dim 1 – 3d	0.05	*
			LV-ChABC vs Naïve	Dim 1 – 3d	0.00007	***
			LV-GFP vs Naïve	Dim 1 – 7d	0.0056	**
			LV-GFP vs LV-ChABC	Dim 1 – 7d	0.0063	##
			Naïve vs LV-GFP	Dim 2 – 6h	0.0012	**
			Naïve vs LV-ChABC	Dim 2 – 6h	0.0068	**
			Naïve vs both treatments	Dim 2 – 12h	0.05	*
			Naïve vs both treatments	Dim 2 – 1d	0.009	**
			LV-ChABC vs Naïve	Dim 2 – 3d	0.05	*
			LV-GFP vs Naïve	Dim 2 – 7d	0.0038	**
			LV-GFP vs LV-ChABC	Dim 2 – 7d	0.0154	#
			LV-GFP vs Naïve	Dim 2 – 14d	0.005	**
			LV-ChABC vs Naïve	Dim 2 – 14d	0.023	*
			LV-ChABC vs Naïve	Dim 3 – 7d	0.0155	*
			Naïve vs both treatments	Dim 3 – 14d	0.001	**
**Extended data Fig 4.**	f	Unpaired *t*-test (two-tailed)	LV-GFP vs LV-ChABC	1d – CXCL1	0.0122	*
			LV-GFP vs LV-ChABC	1d – IL6	0.0004	***
	g	Unpaired *t*-test (two-tailed)	LV-GFP vs LV-ChABC	3d - Leptin	0.0336	*
	h	Unpaired *t*-test (two-tailed)	LV-GFP vs LV-ChABC	7d – CCL3	0.0491	*
			LV-GFP vs LV-ChABC	7d – IL1a	0.0041	**
			LV-GFP vs LV-ChABC	7d – CCL5	0.0022	**
			LV-GFP vs LV-ChABC	7d – IL18	0.05	*
			LV-GFP vs LV-ChABC	7d – IL1b	0.0494	*
**Extended data Fig 5.**	a	Unpaired *t*-test (two-tailed)	LV-GFP vs LV-ChABC	7d - microglia	0.0011	**

	c	Unpaired *t*-test (two-tailed)	LV-GFP vs LV-ChABC	7d - macrophages	0.0029	**
	e	Unpaired *t*-test (two-tailed) Unpaired *t*-test (two-tailed)	LV-GFP vs LV-ChABC	7d - neutrophils	0.0196	*
	b	Unpaired *t*-test (two-tailed)	LV-GFP vs LV-ChABC	7d – MHC-II	0.0137	*
	d	Unpaired *t*-test (two-tailed)	LV-GFP vs LV-ChABC	7d – iNOS	0.0011	**
			LV-GFP vs LV-ChABC	7d – CD68	0.0014	**
			LV-GFP vs LV-ChABC	7d – MHC-II	0.015	*
	f	Unpaired *t*-test (two-tailed)	LV-GFP vs LV-ChABC	3d - leptin	0.0336	*
			LV-GFP vs LV-ChABC	7d – CCL3	0.0491	*
			LV-GFP vs LV-ChABC	7d – IL1a	0.0041	**
			LV-GFP vs LV-ChABC	7d – CCL5	0.0022	**
			LV-GFP vs LV-ChABC	7d – IL18	0.05	*
			LV-GFP vs LV-ChABC	7d – IL1b	0.0494	*
	g	Unpaired *t*-test (two-tailed)	LV-GFP vs LV-ChABC	7d – iNOS	0.0492	*
			LV-GFP vs LV-ChABC	7d – CD68	0.007	**
			LV-GFP vs LV-ChABC	7d – MHC-II	0.0097	**
			LV-GFP vs LV-ChABC	7d – IL1b	0.0024	**
			LV-GFP vs LV-ChABC	7d – TNFa	0.0187	*
			LV-GFP vs LV-ChABC	7d – IL18	0.025	*
			LV-GFP vs LV-ChABC	7d – CCL2	0.0127	*
			LV-GFP vs LV-ChABC	7d – CCL5	0.0378	*
			LV-GFP vs LV-ChABC	7d – CXCL10	0.05	*
**Extended data Fig 6.**	a	Unpaired *t*-test (two-tailed)	LV-GFP vs LV-ChABC	7d – iNOS	0.0492	*
			LV-GFP vs LV-ChABC	7d – CD68	0.007	**
			LV-GFP vs LV-ChABC	7d – MHC-II	0.0097	**
			LV-GFP vs LV-ChABC	7d – IL1b	0.0024	**
			LV-GFP vs LV-ChABC	7d – TNFa	0.0187	*
			LV-GFP vs LV-ChABC	7d – IL18	0.025	*
			LV-GFP vs LV-ChABC	7d – CCL2	0.0127	*
			LV-GFP vs LV-ChABC	7d – CCL5	0.0378	*
			LV-GFP vs LV-ChABC	7d – CXCL10	0.05	*
		Unpaired *t*-test (two-tailed)	LV-GFP vs LV-ChABC	14d – MHC-II	0.009	**
			LV-GFP vs LV-ChABC	14d – CD163	0.03	*
			LV-GFP vs LV-ChABC	14d – CCL5	0.047	*
	b	Unpaired *t*-test (two-tailed)	LV-GFP vs LV-ChABC	14d – MHC-II	0.05	*
	c	Unpaired *t*-test (two-tailed)	LV-GFP vs LV-ChABC	14d – MHC-II	0.015	*

	d	Unpaired *t*-test (two-tailed)	LV-GFP vs LV-ChABC	14d – CD68	0.0141	*
	e	Unpaired *t*-test (two-tailed)	LV-GFP vs LV-ChABC	14d – CD206	0.0147	*
**Extended data Fig 7.**	a	One-way ANOVA	5ug/ml vs control	IL1b	0.0002	***
			5ug/ml vs 1ug/ml	IL1b	0.0063	##
			5ug/ml vs 2.5ug/ml	IL1b	0.047	$
			2.5ug/ml vs control	IL1b	0.0298	*
	b	One-way ANOVA	5ug/ml vs control	iNOS	0.0248	*
	c	One-way ANOVA	5ug/ml vs control	CXCL10	<0.0001	****
			5ug/ml vs 1ug/ml	CXCL10	<0.0001	####
			5ug/ml vs 2.5ug/ml	CXCL10	0.0001	$$$$
			2.5ug/ml vs control	CXCL10	0.0001	***
			2.5ug/ml vs 1ug/ml	CXCL10	0.0035	##
			1ug/ml vs control	CXCL10	0.0079	$$
	d	One-way ANOVA	CSPG Soluble vs control	iNOS	0.024	**
			CSPG Soluble vs CSPG attached	iNOS	0.0305	##
		One-way ANOVA	CSPG Soluble vs control	IL1b	<0.0001	****
			CSPG Soluble vs CSPG attached	IL1b	0.0005	###
			attached vs control	IL1b	0.05	*
		One-way ANOVA	CSPG Soluble vs control	CCL5	<0.0001	****
			CSPG Soluble vs CSPG attached	CCL5	<0.0001	####
	g	One-way ANOVA	M1 vs M0	iNOS/ArgI	0.0049	**
			M1 vs M2	iNOS/ArgI	0.0113	*
		One-way ANOVA	M1 vs M0	IL1b	0.0007	***
			M1 vs M2	IL1b	0.0017	**
		One-way ANOVA	M1 vs M0	CCL5	<0.0001	****
			M1 vs M2	CCL5	<0.0001	****
		One-way ANOVA	M2 vs M0	CD206	0.0021	**
			M2 vs M1	CD206	0.002	**
		One-way ANOVA	M2 vs M0	TGFb	0.0058	**
			M2 vs M1	TGFb	0.0006	***
**Extended data Fig 8.**	b	One-way ANOVA	M1 vs M2	beads	<0.0001	****
			M1+CSPG vs M2	beads	<0.0001	****
			M1 vs M2+CSPG	beads	0.0061	**
			M2 vs M2+CSPG	beads	<0.0001	****
**Extended data Fig 9.**	c	One-way ANOVA	Neonatal microglia vs astrocytes	GFAP	0.0012	**
			Adult microglia vs astrocytes	GFAP	0.0006	***
	d	One-way ANOVA	-CSPG vs +CSPG	IL1b - Macrophages	0.0009	***

			-CSPG vs +CSPG	IL1b – Microglia Nn.	0.0011	**
			-CSPG vs +CSPG	IL1b – Microglia Ad.	0.0336	*
		One-way ANOVA	-CSPG vs +CSPG	iNOS - Macrophages	0.0006	***
			-CSPG vs +CSPG	iNOS – Microglia Nn.	0.0113	*
			-CSPG vs +CSPG	iNOS – Microglia Ad.	0.0284	*
		One-way ANOVA	-CSPG vs +CSPG	IL6 - Macrophages	<0.0001	****
		One-way ANOVA	-CSPG vs +CSPG	CXCL10 - Macrophages	0.0093	**
			-CSPG vs +CSPG	CXCL10 – Microglia Ad.	0.0046	**
		One-way ANOVA	-CSPG vs +CSPG	CCL5 - Macrophages	0.0098	**
			-CSPG vs +CSPG	CCL5 – Microglia Nn.	0.0216	*
		One-way ANOVA	-CSPG vs +CSPG	NfkB - Macrophages	0.0301	*
		One-way ANOVA	-CSPG vs +CSPG	TNFa - Macrophages	0.0217	*
			-CSPG vs +CSPG	TNFa – Microglia Nn.	0.0020	**
			-CSPG vs +CSPG	TNFa – Microglia Ad.	0.0186	*
		One-way ANOVA	-CSPG vs +CSPG	CCL3 - Macrophages	0.0015	**
			-CSPG vs +CSPG	CCL3 – Microglia Nn.	0.00011	***
			-CSPG vs +CSPG	CCL3 – Microglia Ad.	0.0025	**
**Extended data Fig 10.**	b	One-way ANOVA	CSPG vs untreated	IL1b	<0.0001	****
			CSPG vs untreated	iNOS	<0.0001	****
			CSPG vs untreated	IL6	<0.0001	****
			CSPG vs untreated	CXCL10	<0.0001	****
			CSPG vs untreated	CCL2	<0.0001	****
			CSPG vs untreated	CCL3	<0.0001	****
			CSPG vs untreated	CCL5	0.0001	****
			CSPG vs untreated	TNFa	<0.0001	****
			4S-DS vs untreated	CD206	0.0469	#
**Extended data Fig 11.**	a	One-way ANOVA	CSPG vs CSPG+ISP	CD206	0.042	#
	b	One-way ANOVA	CSPG vs CSPG+TAK242	IL1b	0.0048	**
		One-way ANOVA	CSPG vs CSPG+TAK242	iNOS	0.0166	*
		One-way ANOVA	CSPG vs CSPG+TAK242	IL6	<0.0001	****
		One-way ANOVA	CSPG vs CSPG+TAK242	CXCL10	<0.0001	****

		One-way ANOVA	CSPG vs CSPG+TAK242	CCL2	<0.0001	****
		One-way ANOVA	CSPG vs CSPG+TAK242	CCL3	0.001	**
		One-way ANOVA	CSPG vs CSPG+TAK242	CCL5	0.0001	***
		One-way ANOVA	CSPG vs CSPG+TAK242	TNFa	0.0145	*
	c	One-way ANOVA	CSPG vs CSPG+TAK242	IL1b	0.0012	**
		One-way ANOVA	CSPG vs CSPG+TAK242	IL6	<0.0001	****
		One-way ANOVA	CSPG vs CSPG+TAK242	CCL2	0.0006	***
		One-way ANOVA	CSPG vs CSPG+TAK242	CCL3	<0.0001	****
		One-way ANOVA	CSPG vs CSPG+TAK242	TNFa	0.0427	*
		One-way ANOVA	CSPG vs CSPG+TAK242	CD206	0.0432	*
	d	One-way ANOVA	CSPG vs CSPG+TAK242	IL1b	<0.0001	****
		One-way ANOVA	CSPG vs CSPG+ISP	iNOS	0.041	#
			CSPG vs CSPG+TAK242	iNOS	0.05	*
		One-way ANOVA	CSPG vs CSPG+TAK242	IL6	<0.0001	****
		One-way ANOVA	CSPG vs CSPG+TAK242	CXCL10	<0.0001	****
		One-way ANOVA	CSPG vs CSPG+ISP	CCL2	0.003	##
			CSPG vs CSPG+TAK242	CCL2	<0.0001	****
		One-way ANOVA	CSPG vs CSPG+TAK242	CCL3	<0.0001	****
		One-way ANOVA	CSPG vs CSPG+ISP	CCL5	0.04	#
		One-way ANOVA	CSPG vs CSPG+TAK242	CCL5	<0.0001	****
		One-way ANOVA	CSPG vs CSPG+TAK242	TNFa	0.0001	***
**Extended data Fig 12.**	a	Unpaired *t*-test (two-tailed)	M1-like vs M2-like	TLR4	<0.0001	****
	c	Unpaired *t*-test (two-tailed)	M1-like vs M2-like	TLR4	<0.0001	****
	d	Unpaired *t*-test (two-tailed)	M1-like vs M2-like	TLR4	0.0086	**
	f	One-way ANOVA	M1 vs M1 perm	TLR4	<0.0001	****
			M2 vs M1	TLR4	<0.0001	****
			M2 vs M1 perm	TLR4	<0.0001	####
			M2 perm vs M1	TLR4	<0.0001	****
			M2 perm vs M1 perm	TLR4	<0.0001	####
			M2 perm vs M2	TLR4	0.0002	$$$$

	i	Unpaired *t*-test (two-tailed)	CD43- vs CD43+	TLR4	0.0008	***
	j	Unpaired *t*-test (two-tailed)	CD43- vs CD43+	TLR4	0.0435	*
**Extended data Fig 13.**	b	Unpaired *t*-test (two-tailed)	-CSPG vs +CSPG	p-p38/p38	0.0411	*
	d	Unpaired *t*-test (two-tailed)	-CSPG vs +CSPG	p-p38/p38	0.0356	*
	f	One-way ANOVA	M2 vs M1	p-p38/p38	0.0001	***
			M2 vs M1+CSPG	p-p38/p38	0.0001	###
			M2+CSPG vs M1	p-p38/p38	<0.0001	****
			M2+CSPG vs M1+CSPG	p-p38/p38	<0.0001	####
			M2+CSPG vs M2	p-p38/p38	<0.0001	$$$$
	h	One-way ANOVA	CSPG vs CSPG+p38 inh	IL1b	0.008	**
		One-way ANOVA	CSPG vs CSPG+p38 inh	iNOS	0.004	**
		One-way ANOVA	CSPG vs CSPG+p38 inh	IL6	<0.0001	****
		One-way ANOVA	CSPG vs CSPG+p38 inh	CXCL10	0.0009	***
		One-way ANOVA	CSPG vs CSPG+p38 inh	CCL5	0.0006	***
**Extended data Fig 14.**	b	One-way ANOVA	WT vs TLR4-/-	M1	0.0431	*
			WT vs TLR4-/- + CSPG	M1	0.0450	*
			WT + CSPG vs TLR4-/-	M1	0.007	##
			WT + CSPG vs TLR4-/- + CSPG	M1	0.0072	##
		One-way ANOVA	WT vs WT + CSPG	M2	0.0270	*
			WT vs TLR4-/-	M2	0.048	*
			WT + CSPG vs TLR4-/-	M2	0.0326	#
			WT+ CSPG vs TLR4-/- + CSPG	M2	0.0441	#
	d	One-way ANOVA	WT vs TLR4-/-	IL1b	<0.0001	****
			WT vs TLR4-/- + CSPG	IL1b	<0.0001	****
			WT + CSPG vs TLR4-/-	IL1b	<0.0001	$$$$
			WT + CSPG vs TLR4-/- + CSPG	IL1b	<0.0001	$$$$
		One-way ANOVA	WT vs TLR4-/-	iNOS	0.0376	*
			WT vs TLR4-/- + CSPG	iNOS	0.0392	*
			WT + CSPG vs TLR4-/-	iNOS	0.0063	$$
			WT + CSPG vs TLR4-/- + CSPG	iNOS	0.0065	$$
		One-way ANOVA	WT vs TLR4-/- + CSPG	CXCL10	0.0083	**
			WT + CSPG vs TLR4-/-	CXCL10	0.0071	$$
			WT + CSPG vs TLR4-/- + CSPG	CXCL10	0.0191	$
		One-way ANOVA	WT vs TLR4-/- + CSPG	CCL2	0.0322	*
			WT + CSPG vs TLR4-/-	CCL2	0.0030	$$

			WT + CSPG vs TLR4-/- + CSPG	CCL2	0.0238	$
		One-way ANOVA	WT vs TLR4-/-	CCL5	0.0283	*
			WT vs TLR4-/- + CSPG	CCL5	0.0221	*
			WT + CSPG vs TLR4-/-	CCL5	0.0008	$$$
			WT + CSPG vs TLR4-/- + CSPG	CCL5	0.0007	$$$
		One-way ANOVA	WT vs TLR4-/-	MHC-II	0.0045	**
			WT vs TLR4-/- + CSPG	MHC-II	0.0174	*
			WT + CSPG vs TLR4-/-	MHC-II	0.0031	$$
			WT + CSPG vs TLR4-/- + CSPG	MHC-II	0.0116	$
		One-way ANOVA	WT vs TLR4-/-	CD206	<0.0001	****
			WT vs TLR4-/- + CSPG	CD206	<0.0001	****
			WT + CSPG vs TLR4-/-	CD206	<0.0001	$$$$
			WT + CSPG vs TLR4-/- + CSPG	CD206	<0.0001	$$$$
	e	One-way ANOVA	WT vs WT + CSPG	IL1b	0.0085	**
			WT + CSPG vs TLR4-/-	IL1b	0.0083	##
			WT + CSPG vs TLR4-/- + CSPG	IL1b	0.0247	&
		One-way ANOVA	WT vs WT + CSPG	iNOS	0.0270	*
			WT + CSPG vs TLR4-/-	iNOS	0.0090	##
			WT + CSPG vs TLR4-/- + CSPG	iNOS	0.0087	&&
		One-way ANOVA	WT vs WT + CSPG	IL6	0.0073	**
			WT + CSPG vs TLR4-/-	IL6	0.0114	#
			WT + CSPG vs TLR4-/- + CSPG	IL6	0.0049	&&
		One-way ANOVA	WT vs WT + CSPG	CXCL10	0.0406	*
			WT vs TLR4-/-	CXCL10	0.0497	#
			WT vs TLR4-/- + CSPG	CXCL10	0.026	*
			TLR4-/- vs TLR4-/- + CSPG	CXCL10	0.0292	#
		One-way ANOVA	WT vs WT + CSPG	CCL2	0.0005	***
			WT + CSPG vs TLR4-/-	CCL2	0.001	###
			WT + CSPG vs TLR4-/- + CSPG	CCL2	0.0013	&&
		One-way ANOVA	WT vs WT + CSPG	CCL5	0.0117	*
			WT + CSPG vs TLR4-/-	CCL5	0.0018	###
			WT + CSPG vs TLR4-/- + CSPG	CCL5	0.0042	&&
		One-way ANOVA	WT vs WT + CSPG	TNFa	0.0001	***
			WT + CSPG vs TLR4-/-	TNFa	0.0001	###
			WT + CSPG vs TLR4-/- + CSPG	TNFa	0.0003	&&&
	f	One-way ANOVA	WT vs WT + CSPG	IL1b	0.0047	**
			WT + CSPG vs TLR4-/-	IL1b	0.0022	##
			WT + CSPG vs TLR4-/- + CSPG	IL1b	0.0023	&&
		One-way ANOVA	WT vs WT + CSPG	CXCL10	<0.0001	****
			WT + CSPG vs TLR4-/-	CXCL10	<0.0001	####

			WT + CSPG vs TLR4-/- + CSPG	CXCL10	<0.0001	&&&&
		One-way ANOVA	WT vs TLR4-/-	CCL2	0.0103	*
			WT + CSPG vs TLR4-/- + CSPG	CCL2	0.0136	$
			WT + CSPG vs TLR4-/-	CCL2	0.0295	*
			WT + CSPG vs TLR4-/- + CSPG	CCL2	0.0443	$
		One-way ANOVA	WT vs TLR4-/-	CCL5	0.0004	***
			WT + CSPG vs TLR4-/- + CSPG	CCL5	0.0004	$$$
			WT + CSPG vs TLR4-/-	CCL5	0.0009	***
			WT + CSPG vs TLR4-/- + CSPG	CCL5	0.0008	$$$
		One-way ANOVA	WT vs WT + CSPG	TNFa	<0.0001	****
			WT + CSPG vs TLR4-/-	TNFa	<0.0001	####
			WT + CSPG vs TLR4-/- + CSPG	TNFa	<0.0001	&&&&
	g	Unpaired *t*-test (two-tailed)	WT vs TLR4-/-	M1 - TLR2	<0.0001	****
	h	Unpaired *t*-test (two-tailed)	WT vs TLR4-/-	M2 - TLR2	0.0002	***
		Unpaired *t*-test (two-tailed)	WT vs TLR4-/-	M2 - TLR6	0.0146	*
**Extended data Fig 15.**	b	One-way ANOVA	WT vs WT+CSPG	Axonal length	0.0001	***
			WT+CSPG vs WT+CSPG+ChABC	Axonal length	0.0156	#
		One-way ANOVA	TLR4-/- vs TLR4-/- +CSPG	Axonal length	0.0001	***
			TLR4-/- +CSPG vs TLR4-/- +CSPG+ChABC	Axonal length	0.0006	##
			TLR4-/- vs TLR4-/- +CSPG+ChABC	Axonal length	0.013	*
