# Supplementary material for: Chondroitin sulfate proteoglycans prevent immune cell phenotypic conversion and inflammation resolution via TLR4 in rodent models of spinal cord injury
Source: Nat Commun. 2022 May 25;13:2933. doi: 10.1038/s41467-022-30467-5 (PMC9133109; doi:10.1038/s41467-022-30467-5)
Supplement: Supplementary file 2 — Reporting Summary [file 41467_2022_30467_MOESM2_ESM.pdf]

nature portfolio

Corresponding author(s): Elizabeth J. Bradbury
Last updated by author(s): Mar 8, 2022

Reporting Summary

Nature Portfolio wishes to improve the reproducibility of the work that we publish. This form provides structure for consistency and transparency in reporting. For further information on Nature Portfolio policies, see our Editorial Policies and the Editorial Policy Checklist.

Statistics

For all statistical analyses, confirm that the following items are present in the figure legend, table legend, main text, or Methods section.

n/a	Confirmed	
☐	☒	The exact sample size (n) for each experimental group/condition, given as a discrete number and unit of measurement
☐	☒	A statement on whether measurements were taken from distinct samples or whether the same sample was measured repeatedly
☐	☒	The statistical test(s) used AND whether they are one- or two-sided<br>*Only common tests should be described solely by name; describe more complex techniques in the Methods section.*
☐	☒	A description of all covariates tested
☐	☒	A description of any assumptions or corrections, such as tests of normality and adjustment for multiple comparisons
☐	☒	A full description of the statistical parameters including central tendency (e.g. means) or other basic estimates (e.g. regression coefficient) AND variation (e.g. standard deviation) or associated estimates of uncertainty (e.g. confidence intervals)
☐	☒	For null hypothesis testing, the test statistic (e.g. F, t, r) with confidence intervals, effect sizes, degrees of freedom and P value noted<br>*Give P values as exact values whenever suitable.*
☒	☐	For Bayesian analysis, information on the choice of priors and Markov chain Monte Carlo settings
☒	☐	For hierarchical and complex designs, identification of the appropriate level for tests and full reporting of outcomes
☐	☒	Estimates of effect sizes (e.g. Cohen's d, Pearson's r), indicating how they were calculated

Our web collection on statistics for biologists contains articles on many of the points above.

Software and code

Policy information about availability of computer code

Data collection

Imaging:
LSM710 confocal microscope
Nikon A1R Si Confocal Imaging system on an Eclipse Ti-E inverted microscope
Apotome Zeiss microscope
ZEISS Imager Z1 fluorescence microscope equipped with a AxioCam MRm camera

Flow cytometry:
LSRFortessa III flow cytometer (BD)
FACSymphony A5 flow cytometer (BD)
FACSAria II (BD)

Immunoblotting:
Kodak processor and Alliance Q9 Advanced (Uvitec Cambridge)

Luminex:
Luminex xPONENT

Gene expression:
RNA concentration and integrity were determined by a NanoDrop ND-1000 spectrophotometer (Thermo Scientific).
LightCycler 480 (Roche, Switzerland)

Data analysis

All softwares and software version used to analyse data are described in the Method section at the relevant paragraph:

Flow cytometry data was analysed with FlowJo plugin (version v10- Treestar) and tSNE plots using FlowJo plugin (version v10-LLC) and clustered by relative marker expression into nodes using the following parameters: perplexity=50, theta=0.5 and 500 iterations.

For histology analysis, tile scans were stitched in post-acquisition processing using AxioVision 4.8.2.0 software. Staining was quantified using ZEISS ZEN lite 3.4 (.NET Framework 4.0.30319.472000) software.

GIMP is 2.10.22 for figure and supplementary figures design.

Image J (v1.51j) for Image analysis.

Cytokine protein levels were analysed using the MILLIPLEX MAP Rat 27 Cytokine & Chemokine magnetic bead panel (RECYMAG65K27PMX - Millipore)

LightCycler® 480 Software (v1.5.1.62) for gene expression quantification.

Dual multiple factor analysis (dMFA) was conducted to study the longitudinal evolution of the multidimensional gene expression profile using the R package actoMineR (version 2.3). Loading and score plots were generated using the ggplot2 R package.

Statistical analyses were performed with GraphPad Prism v8 and v9 software.

For manuscripts utilizing custom algorithms or software that are central to the research but not yet described in published literature, software must be made available to editors and reviewers. We strongly encourage code deposition in a community repository (e.g. GitHub). See the Nature Portfolio guidelines for submitting code & software for further information.

Data

Policy information about availability of data

All manuscripts must include a data availability statement. This statement should provide the following information, where applicable:

- Accession codes, unique identifiers, or web links for publicly available datasets
- A description of any restrictions on data availability
- For clinical datasets or third party data, please ensure that the statement adheres to our policy

All data generated are included in this article (main or supplementary information files) and are provided in the Source Data file. Additional information can be obtained from the corresponding author. Source data are provided with this paper.

Field-specific reporting

Please select the one below that is the best fit for your research. If you are not sure, read the appropriate sections before making your selection.

☒ Life sciences ☐ Behavioural & social sciences ☐ Ecological, evolutionary & environmental sciences

For a reference copy of the document with all sections, see nature.com/documents/nr-reporting-summary-flat.pdf

Life sciences study design

All studies must disclose on these points even when the disclosure is negative.

Sample size

Sample sizes were determined based on power calculations (G*) and based on previous reports.

For immune cell recruitment study by flow cytometry n = 9 per group at 1 dpi; n = 12 at 3 dpi; n = 12 at 7 dpi; n = 6 at 14 dpi; n = 4 at 28 dpi. t-SNE analysis at day 7 after SCI was performed with n = 4 per treatment.

For dynamic inflammatory gene expression analysis n = 6 for each group-time combination except LV-GFP at 12h, LV-ChABC at 6h and 12h post injury (n = 4); LV-GFP at 3 dpi (n = 5) and LV-ChABC at 7 dpi (n = 7). Same samples were used for Cytokine protein level comparison between LV-GFP and LV-ChABC treatment groups assessed by Luminex analysis. Deeper inflammatory gene expression was performed at 7dpi with n=6 naive animals and n=5 per treatment.

For histology analysis n=3 in LV-ChABC and n=4 in LV-GFP groiups.

In vitro first screening experiments to evaluate immunomodulatory role of CSPG in microglial cells and BMDM n=3 per condition and cell population except CSPG digestion experiments where n=4 per treatment and cell population. Then to evaluate in depth the immunomodulatory role of CSPG, its digestion products and the effect of TLR4, PTPsigma and p38 inhibitors; n=12 in control and CSPG groups, n=5 in disaccharide assay and n = 4 in each inhibitor group. CSPG effect in WT and TLR4 KO mice n=3.

Phagocytosis assay was evaluated in two different batches analyzing 5 randomly distributed frames per coverslip in a total of 2 coverslips per treatment and batch.

	For TLR4 expression analysis n= 12 per group in M1-like vs M2-like in rat BMDM; n= 4 per group in M1-like vs M2-like in mouse BMDM; n = 3 per group in CD43- vs CD43+.<br><br>For inflammatory pathway activation assessment by Western blot n=3 per group and time point. For fluorescence analysis at least 80 cells in each batch (2 different batches) analysed for each treatment.<br><br>Finally for CSPG effect on neurite outgrowth, in each batch between 120-175 neurons were analysed per condition, in triplicates in a total of 2 different batches.
Data exclusions	Animals with an injury force that fell out of the range +/- 10% expected force (kDyne) were excluded.
Replication	In vitro, in vivo flow cytometry and in vivo gene expression experiments were successful in two/three different experiments/cohort and data is accumulated during the analysis process.
Randomization	During surgical procedures blinding was ensured by the experimenter injecting the vectors being unaware of animal identification number. Animals across both groups were randomized into cages, where an experimenter unaware of treatment group randomly assigned caging.<br><br>In vitro experiments in rat, cells were randomly polarized and activated from the same pool of cells. Finally, in case of in vitro experiments in mice to evaluate the role of TLR4 for obvious reasons one experimenter was aware of which cells were WT or KO for TLR4. However, a second experimenter was blind to polarize, activate and analyse the effect of the treatments in each cell population.
Blinding	Experimenters were blinded during in vivo procedures such as surgeries, lentiviral injections or tissue processing.<br><br>During in vitro experiments, activation, data collection and all statistical analysis was completed with the investigator blind to the experimental coding. In some cases such as TLR4 WT vs KO experiments, blinding was not possible during cell isolation. However, as in rest of in vitro experiments, activation, data collection and all statistical analysis was completed with the investigator blind to the experimental coding.

Reporting for specific materials, systems and methods

We require information from authors about some types of materials, experimental systems and methods used in many studies. Here, indicate whether each material, system or method listed is relevant to your study. If you are not sure if a list item applies to your research, read the appropriate section before selecting a response.

Materials & experimental systems

n/a	Involved in the study	
☐	☒	Antibodies
☐	☒	Eukaryotic cell lines
☒	☐	Palaeontology and archaeology
☐	☒	Animals and other organisms
☒	☐	Human research participants
☒	☐	Clinical data
☒	☐	Dual use research of concern

Methods

n/a	Involved in the study	
☒	☐	ChIP-seq
☐	☒	Flow cytometry
☒	☐	MRI-based neuroimaging

Antibodies

Antibodies used	All antibodies and dilutions are listed in Article Methods:<br><br>Flow cytometry:<br><br>CD45 BUV395 - BDBioscience-740258 - Dilution 1:250<br>CD11b V450 - BDBioscience-562108 - Dilution 1:300<br>CD43 PE-Cy7 - Miltenyi Biotec - 130-107-721 - Dilution 1:150<br>HIS48 ThermoFisher - 14-0570-82* - Dilution 1:200<br>* AF700 kit (ab269824)<br>CD45RA BUV805 - BDBioscience-741973 - Dilution 1:250<br>CD3 BV605 - BDBioscience-563949 - Dilution 1:250<br>CD4 BUV737 - BDBioscience-749058 - Dilution 1:250<br>CD8 BUV563 - BDBioscience-748879 - Dilution 1:250<br>CD68 APC-Cy7 - Miltenyi Biotec - 130-103-366 - Dilution 1:125<br>CD86 BV510 - BDBioscience- 743212 - Dilution 1:250<br>INOS AF647 - Santa Cruz - sc-7271 AF647 - Dilution 1:300<br>MHC II PerCP-Cy5.5 - Miltenyi Biotec - 130-107-877 - Dilution 1:150<br>Arg I - Santa Cruz - sc-271430* - Dilution 1:200<br>* PE-Cy5 kit (ab102893)<br>CD206 FITC - Bioss – bs-4727R-FITC - Dilution 1:150

CD163 PE - GeneTex - GTX42934 - Dilution 1:300
CD4 PE - BDBioiscience - 551397 - Dilution 1:200
CD8 FITC - Miltenyi Biotec – 130-119-664 - Dilution 1:150
RP-1 BV786 - BD Bioscience - 743058 - Dilution 1:200
TLR4 AF647 - Biotechne - NBP2-27149 - Dilution 1:300
Live/Dead Yellow (BV570) - Biolegend - 423103 - Dilution 1:1000
Live/Dead NIR (APC-Cy7) - Biolegend - 423105 - Dilution 1:1000

Western blot:

Rabbit anti rat p-JNK Cell Signalling – cs3033T - Dilution 1:1000
Mouse anti rat JNK Santa Cruz – sc-7345 - Dilution 1:300
Rabbit anti rat p-ERK 1/2 Cell Signalling – cs9106S - Dilution 1:1000
Mouse anti rat ERK 1/2 Santa Cruz – sc-514302 - Dilution 1:300
Rabbit anti rat p-p38 Cell Signalling – cs4511T - Dilution 1:1000
Mouse anti rat p38 Santa Cruz – sc-7972 - Dilution 1:300
Rabbit anti rat p-p65 Cell Signalling – cs3033T - Dilution 1:1000
Mouse anti rat p65 Cell Signalling – cs6956T - Dilution 1:1000
Rabbit anti !-Actin Abcam – ab179467 - Dilution 1:3000
Rabbit anti rat GAPDH Abcam – ab181602 - Dilution 1:2000
Rabbit anti mouse iNOS Abcam - ab15323 - Dilution 1:600
Mouse anti- a-Actin Sigma – A1978 - Dilution 1:5000

Immunocytochemistry:

Mouse anti-CSPG - AbD Serotec - Dilution 1:250
chicken anti-GFP - Abcam ab13970 - Dilution 1:1000
Mouse anti- CS-56 - Sigma C8035 - Dilution 1:300
Mouse anti-GFAP-488 - EMD Millipore MAB3402X - Dilution 1:500
Rabbit anti-CD206 - Abcam ab64693 - Dilution 1:300
Rabbit anti-iNOS - Abcam ab15323 - Dilution 1:100
Mouse anti-NFH-488 - EMD Millipore MAB5256X - Dilution 1:500
Mouse anti-TLR4 -Santa Cruz sc-293072 - Dilution 1:50
Mouse anti-β-III-tubulin - Neuromics MO15013 - Dilution 1:500
Rabbit anti-p-p38 - Cell Signalling cs9211 - Dilution 1:100
Rabbit anti-P38 - Cell Signalling cs9212 - Dilution 1:100

Validation

Commercial antibodies are validated by the manufacturer. Detailed validation analysis and relevant literatures are provided on the company website for the products used in this study:

Flow cytometry:

- CD45 BUV395 - BDBioscience-740258 - https://www.bdbiosciences.com/en-gb/products/reagents/flow-cytometry-reagents/research-reagents/single-color-antibodies-ruo/buv395-mouse-anti-rat-cd45.740258
- CD11b V450 - BDBioscience-562108 - https://www.bdbiosciences.com/en-gb/products/reagents/flow-cytometry-reagents/research-reagents/single-color-antibodies-ruo/v450-mouse-anti-rat-cd11b.562108
- CD43 PE-Cy7 - Miltenyi Biotec - 130-107-721 - https://www.miltenyibiotec.com/GB-en/products/cd43-antibody-anti-rat-reafinity-rea503.html#pe-vio-770:30-ug-in-1-ml
- HIS48 ThermoFisher - 14-0570-82* - https://www.thermofisher.com/antibody/product/Granulocyte-Marker-Antibody-clone-HIS48-Monoclonal/14-0570-82
 * AF700 kit (ab269824)
- C D45RA BUV805 - BDBioscience-741973 - https://www.bdbiosciences.com/en-gb/products/reagents/flow-cytometry-reagents/research-reagents/single-color-antibodies-ruo/buv805-mouse-anti-rat-cd45ra.741973
- CD3 BV605 - BDBioscience-563949 - https://www.bdbiosciences.com/en-gb/products/reagents/flow-cytometry-reagents/research-reagents/single-color-antibodies-ruo/bv605-mouse-anti-rat-cd3.563949
- CD4 BUV737 - BDBioscience-749058 - https://www.bdbiosciences.com/en-gb/products/reagents/flow-cytometry-reagents/research-reagents/single-color-antibodies-ruo/buv737-mouse-anti-rat-cd4.749058
- CD8 BUV563 - BDBioscience-748879 - https://www.bdbiosciences.com/en-gb/products/reagents/flow-cytometry-reagents/research-reagents/single-color-antibodies-ruo/buv563-mouse-anti-rat-cd8b.748879
- CD68 APC-Cy7 - Miltenyi Biotec - 130-103-366 - https://www.miltenyibiotec.com/GB-en/products/cd68-antibody-anti-rat-reafinity-rea237.html#apc-vio-770:30-ug-in-200-ul
- CD86 BV510 - BDBioscience- 743212 - https://www.bdbiosciences.com/en-gb/products/reagents/flow-cytometry-reagents/research-reagents/single-color-antibodies-ruo/bv510-mouse-anti-rat-cd86.743212
- INOS AF647 - Santa Cruz - sc-7271 AF647 - https://www.scbt.com/es/p/nos2-antibody-c-11
- MHC II PerCP-Cy5.5 - Miltenyi Biotec - 130-107-877 - https://www.miltenyibiotec.com/GB-en/products/mhc-class-ii-i-ek-antibody-anti-mouse-rat-reafinity-rea510.html#percp-vio-700:30-ug-in-1-ml
- Arg I - Santa Cruz - sc-271430* - https://www.scbt.com/p/arginase-1-antibody-e-2?requestFrom=search
 * PE-Cy5 kit (ab102893)

- CD206 FITC - Bioss – bs-4727R-FITC - https://www.biossantibodies.com/datasheets/bs-4727R-FITC
- CD163 PE - GeneTex - GTX42934- https://www.genetex.com/Product/Detail/CD163-antibody-2A10-11-PE/GTX42934
- CD4 PE - BDBioiscience - 551397 - https://www.bdbiosciences.com/en-gb/products/reagents/flow-cytometry-reagents/research-reagents/single-color-antibodies-ruo/pe-mouse-anti-rat-cd4.551397
- CD8 FITC - Miltenyi Biotec – 130-119-664 - https://www.miltenyibiotec.com/GB-en/products/cd8a-antibody-anti-rat-reafinity-rea437.html#pe-vio-770:30-ug-in-1-ml
- RP-1 BV786 - BD Bioscience - 743058 - https://www.bdbiosciences.com/en-gb/products/reagents/flow-cytometry-reagents/research-reagents/single-color-antibodies-ruo/bv786-mouse-anti-rat-rp-1-antigen.743058
- TLR4 AF647 - Biotechne - NBP2-27149 - https://www.novusbio.com/products/tlr4-antibody-76b3571_nbp2-27149af647
- Live/Dead Yellow (BV570) - Biolegend - 423103 - https://www.biolegend.com/en-gb/products/zombie-yellow-fixable-viability-kit-8514?GroupID=BLG2181
- Live/Dead NIR (APC-Cy7) - Biolegend - 423105 - https://www.biolegend.com/en-gb/products/zombie-nir-fixable-viability-kit-8657?GroupID=BLG2181

Western blot:

- Rabbit anti rat p-JNK Cell Signalling – cs3033T - https://www.cellsignal.com/products/primary-antibodies/phospho-nf-kb-p65-ser536-93h1-rabbit-mab/3033
- Mouse anti rat JNK Santa Cruz – sc-7345 - https://www.scbt.com/p/jnk-antibody-d-2?requestFrom=search
- Rabbit anti rat p-ERK 1/2 Cell Signalling – cs9106S - https://www.cellsignal.com/products/primary-antibodies/phospho-p44-42-mapk-erk1-2-thr202-tyr204-e10-mouse-mab/9106
- Mouse anti rat ERK 1/2 Santa Cruz – sc-514302 - https://www.scbt.com/p/erk-1-2-antibody-c-9?requestFrom=search
- Rabbit anti rat p-p38 Cell Signalling – cs4511T - https://www.cellsignal.com/products/primary-antibodies/phospho-p38-mapk-thr180-tyr182-d3f9-xp-rabbit-mab/4511
- Mouse anti rat p38 Santa Cruz – sc-7972 - https://www.scbt.com/p/p38alpha-beta-antibody-a-12?requestFrom=search
- Rabbit anti rat p-p65 Cell Signalling – cs3033T - https://www.cellsignal.com/products/primary-antibodies/phospho-nf-kb-p65-ser536-93h1-rabbit-mab/3033
- Mouse anti rat p65 Cell Signalling – cs6956T - https://www.cellsignal.com/products/primary-antibodies/nf-kb-p65-l8f6-mouse-mab/6956
- Rabbit anti Actin Abcam – ab179467 - https://www.abcam.com/actin-antibody-epr16769-ab179467.html
- Rabbit anti rat GAPDH Abcam – ab181602 - https://www.abcam.com/gapdh-antibody-epr16891-loading-control-ab181602.html
- Rabbit anti mouse iNOS Abcam - ab15323 - https://www.abcam.com/inos-antibody-ab15323.html
- Mouse anti- b-Actin Sigma – A1978 - https://www.sigmaaldrich.com/GB/en/product/sigma/a1978?gclid=Cj0KCQiA95aRBhCsARIsAC2xvfxG6CJmvFB__T5lfEe6uTyL4pIBHTouW23-qdHnda-QaL7jogRWVmoaAu7TEALw_wcB

Immunocytochemistry:

- Mouse anti-CSPG - AbD Serotec - XXXXXXXXXXXXX
- Chicken anti-GFP - Abcam ab13970 - https://www.abcam.com/gfp-antibody-ab13970.html
- Mouse anti- CS-56 - Sigma C8035 - https://www.sigmaaldrich.com/GB/en/product/sigma/c8035
- Mouse anti-GFAP-488 - EMD Millipore MAB3402X - https://www.merckmillipore.com/ES/es/product/Anti-Glial-Fibrillary-Acidic-Protein-Antibody-clone-GA5-Alexa-Fluor-488,MM_NF-MAB3402X?ReferrerURL=https%3A%2F%2Fwww.google.com%2F
- Rabbit anti-CD206 - Abcam ab64693 - https://www.abcam.com/mannose-receptor-antibody-ab64693.html
- Rabbit anti-iNOS - Abcam ab15323 - https://www.abcam.com/inos-antibody-ab15323.html
- Mouse anti-NFH-488 - EMD Millipore MAB5256X - https://www.merckmillipore.com/ES/es/product/Anti-Neurofilament-H-Antibody-clone-NE14-Alexa-Fluor-488-Conjugated,MM_NF-MAB5256X
- Mouse anti-TLR4 -Santa Cruz sc-293072 - https://www.scbt.com/p/tlr4-antibody-25?requestFrom=search
- Mouse anti-β-III-tubulin - Neuromics MO15013 - https://www.neuromics.com/MO15013
- Rabbit anti-p-p38 - Cell Signalling cs9211 - https://www.cellsignal.com/products/primary-antibodies/phospho-p38-mapk-thr180-tyr182-antibody/9211
- Rabbit anti-P38 - Cell Signalling cs9212 - https://www.cellsignal.com/products/primary-antibodies/p38-mapk-antibody/9212

Eukaryotic cell lines

Policy information about cell lines

Cell line source(s)	Source:HEK293T cells: ATCC
Authentication	Authentication: HEK293T cells: ATCC Cell line authentication service. Report attached in Suplementary information
Mycoplasma contamination	Not tested
Commonly misidentified lines (See ICLAC register)	None used

Animals and other organisms

Policy information about studies involving animals; ARRIVE guidelines recommended for reporting animal research

Laboratory animals	One hundred and eigthy-two (182) adult female Lister Hooded (LH) rats (200–220 g; Charles River) were used for in vivo and in vitro studies.

Twenty-four (24) adult female mice C57/BL6 wild-type (WT; n=8) and TLR4-Knock-out (TLR4-/-; n=8) (23-25 g; C57/BL6 background kindly provided by Dr. S. Akira, Osaka, Japan) were used for in vitro experiments.

Wild animals	The study did not involve wild animals.
Field-collected samples	The study did not involve samples collected from the field.
Ethics oversight	In rats, all procedures were performed in accordance with the United Kingdom Animals (Surgical Procedures) Act 1986, approved by the Animal Welfare and Ethical Review Body (AWERB) of King's College London and conducted under Home Office Project License PEE6F3C82.<br><br>In mice, all procedures were carried out in accordance with the guidelines approved by the European Communities Council Directive (86/609/ECC) and by Spanish Royal Decree 1201/2005 with the approval of the Ethical Committee of Animal Experimentation of the Príncipe Felipe Research Centre (Valencia, Spain).

Note that full information on the approval of the study protocol must also be provided in the manuscript.

Flow Cytometry

Plots

Confirm that:

- [x] The axis labels state the marker and fluorochrome used (e.g. CD4-FITC).
- [x] The axis scales are clearly visible. Include numbers along axes only for bottom left plot of group (a 'group' is an analysis of identical markers).
- [x] All plots are contour plots with outliers or pseudocolor plots.
- [x] A numerical value for number of cells or percentage (with statistics) is provided.

Methodology

Sample preparation	To study the dynamics of immune cells after SCI, spinal cords from sham (laminectomy only) and injured LV-GFP or LV-ChABC treated rats were harvested at day 1, 3, 7, 14 and 28 days after lesion. Animals were deeply anaesthetized with sodium pentobarbital (Euthatal®, 80 mg/kg, administered intraperitoneally) and transcardially perfused with ice-cold 1X phosphate buffered saline (PBS) + 2% EDTA. Immediately after perfusion, 8 mm of the injured spinal cord centered around the lesion epicentre was dissected and placed into ice-cold PBS. Tissue was mechanically dissociated and then passed through a 70 "m cell strainer (BD Falcon, Germany), and centrifuged at 300 g at 4 °C. The pellet was incubated with Myelin Removal Beads II (Miltenyi Biotec, Germany) and passed through LS columns (Miltenyi Biotech) to elute cells.<br><br>For primary cell cultures, cells were detached by incubating with Enzyme Free cell dissociation buffer (Milipore). Remaining adherent cells were gently scraped and passed through a 70 "m cell strainer (BD Falcon, Germany). After centrifugation, cells were resuspended in DPBS (Gibco).
Instrument	Cells were analyzed using an LSRFortessa III flow cytometer (BD) and FACSymphony A5 (BD). Cells were isolated using an Aria III (BD).
Software	Flow cytometry data was analyzed with FlowJo (V10, Treestar) software.<br>t-SNE analysis was performed by t-SNE FlowJo Plugin plugin (version v10-LLC) .
Cell population abundance	To study the dynamics of immune cells after SCI the entire epicentre was analyzed. Number of CD45+ cells analyzed depends on the time point that was assessed (numbers change from acute to sub-acute time points).<br><br>For cell cultures, at least 10.000 CD11b+ Cd45+ cells were analyzed.
Gating strategy	Gating strategy is represented in Supplementary information (Methods, extended data Fig. 1 and figure 5).<br>Fluorescence minus one (FMO) experiment and isotype-matched control samples were run prior to this study to establish the positiveness of the samples.<br><br>Single live cells were gated on the basis of dead cell exclusion (L/D), side (SSC-A) and forward scatter (FSC-A) gating, and doublet exclusion using side scatter width (SSC-W) against SSC-A. To perform the analysis, cells were first gated for CD45 to ensure that only infiltrating leukocytes and resident microglia were selected. Then, a combination of markers were used to identify the following cell populations: microglia (CD45+medium, CD11b+medium/high, CD3 and CD19low and SSClow/medium), macrophages (CD45+high, CD11b+high, CD3 and CD19low, and SSClow/medium), Neutrophils (CD45+high, CD11b+medium, CD43+high, HIS-48+, RP1+, CD3 and CD19low and SSChigh), CD4+ (CD45+high, CD11b-or low, CD3-, CD4+, CD8-), CD8+ T cells (CD45+high, CD11b- or low, CD3+, CD4-, CD8+) and B cells (CD45+high, CD11b- or low, CD11c-, CD3-, CD45RA+). To study the phenotype of microglia and macrophages, in addition to prior described antibodies, these cells were further differentiated based on CD86, CD68, MHC II, iNOS, CD43, HIS48, CD163, CD206, and Arg1 expression.<br><br>The complex maps of immune cells were plotted by t-Distributed Stochastic Neighbour Embedding (t-SNE). Concatenating

graphs are generated from all samples in each group. Manually-gated viable CD45+ leukocytes were overlaid into the tSNE plots using FlowJo plugin (version v10-LLC) and clustered by relative marker expression into nodes using the following parameters: perplexity=50, theta=0.5 and 500 iterations.

Cell Sorting. Extracellular staining was performed in 7 dpi spinal cord isolated cell samples. After Fc-receptor blockade and live/dead staining, specific antibodies were used to identify neutrophils, microglia, CD43-/CD43+ monocyte/macrophages and T CD4 populations (antibodies detailed in Supplementary Table 1).

For cell culture analysis, single live cells were ated on the basis of dead cell exclusion (L/D), side (SSC-A) and forward scatter (FSC-A) gating, and doublet exclusion using side scatter width (SSC-W) against SSC-A. To perform the analysis, cells were gated for CD45 and CD11b+ to ensure that only macrophages were selected.

☒ Tick this box to confirm that a figure exemplifying the gating strategy is provided in the Supplementary Information.
